# Recurrent somatic mutations as predictors of immunotherapy response

**DOI:** 10.1038/s41467-022-31055-3

**Published:** 2022-07-08

**Authors:** Zoran Z. Gajic, Aditya Deshpande, Mateusz Legut, Marcin Imieliński, Neville E. Sanjana

**Affiliations:** 1 New York Genome Center, New York, NY 10013 USA; 2 Department of Biology, New York University, New York, NY 10002 USA; 3 Department of Neuroscience and Physiology, New York University School of Medicine, New York, NY 10016 USA; 4 Tri-institutional Ph.D. Program in Computational Biology and Medicine, New York, NY USA; 5 Department of Pathology and Laboratory Medicine, Englander Institute for Precision Medicine, Institute for Computational Biomedicine, and Meyer Cancer Center, Weill Cornell Medicine, New York, NY 10065 USA

**Keywords:** Cancer genetics, Predictive medicine

## Abstract

Immune checkpoint blockade (ICB) has transformed the treatment of metastatic cancer but is hindered by variable response rates. A key unmet need is the identification of biomarkers that predict treatment response. To address this, we analyzed six whole exome sequencing cohorts with matched disease outcomes to identify genes and pathways predictive of ICB response. To increase detection power, we focus on genes and pathways that are significantly mutated following correction for epigenetic, replication timing, and sequence-based covariates. Using this technique, we identify several genes (*BCLAF1, KRAS, BRAF*, and *TP53)* and pathways (MAPK signaling, p53 associated, and immunomodulatory) as predictors of ICB response and develop the Cancer Immunotherapy Response CLassifiEr (CIRCLE). Compared to tumor mutational burden alone, CIRCLE led to superior prediction of ICB response with a 10.5% increase in sensitivity and a 11% increase in specificity. We envision that CIRCLE and more broadly the analysis of recurrently mutated cancer genes will pave the way for better prognostic tools for cancer immunotherapy.

## Abstract (web summary)

Few genetic biomarkers are known for cancer immunotherapy. Here the authors identify recurrently-mutated genes and pathways associated with treatment response and develop a classifier using tumour whole exome sequencing and clinical features.

## Introduction

Immunotherapies, such as immune checkpoint blockade (ICB) have transformed the treatment of advanced-stage cancers^1^. Patients with unresectable or metastatic disease can survive many years with ICB treatment^2^, although only a minority of treated patients demonstrate durable responses^3^. Given the high cost and potential toxicity of these drugs, a major unmet need in immuno-oncology is a robust and clinically practical algorithm to predict ICB response.

Currently, there are several biomarkers that positively correlate with ICB response, such as patient age^4^, tumor type^5^, and tumor mutational burden (TMB)^6^. TMB, which is generally calculated from targeted gene or exome sequencing data, is the most well-established marker of ICB response^6–11^ and is used in an FDA-approved clinical diagnostic (FoundationOne CDx). TMB-high tumors are thought to be more immunogenic and hence responsive to ICB due to their increased burden of neoantigens.

Previous studies have proposed RNA-based biomarkers of ICB response based on the expression levels of immune checkpoint^12^ and T-cell associated^13^ genes; although these present different challenges for routine clinical use, as RNA is more labile and prone to degradation than DNA. Immunohistochemistry-based assessment of PD-L1 expression is routinely applied in the clinic, but has shown inconsistent correlation with ICB response^14^. Though recent whole exome sequencing (WES) studies have attempted to go beyond TMB to link specific DNA alterations to ICB response^7,15–19^, they have been limited by low sample sizes and underpowered (genome-wide) analytic approaches.

Here, we combine six cohorts with Response Evaluation Criteria in Solid Tumors (RECIST) characterization and matched WES for 319 patients across a variety of tumor types with the goal of identifying gene and pathway biomarkers of ICB response. Although we build a larger cohort by pooling several studies, the sample size is still limiting for genome-wide significance. To address this, we focused on recurrently mutated (and likely positively selected) genes and pathways, which we nominated after correcting for known covariates of neutral mutation density^20^. We then determined the ability of these genes and pathways to predict response using a simple logistic regression model. These features were combined with other predictive variables such as age, tumor type and TMB, to create the Cancer Immunotherapy Response CLassifiEr (CIRCLE), which outperformed current TMB-based biomarkers such as the FoundationOne CDx^11^.

## Results

### The aggregated cohort replicates known predictors of ICB response such as tumor mutational burden and age

**Fig. 1:**
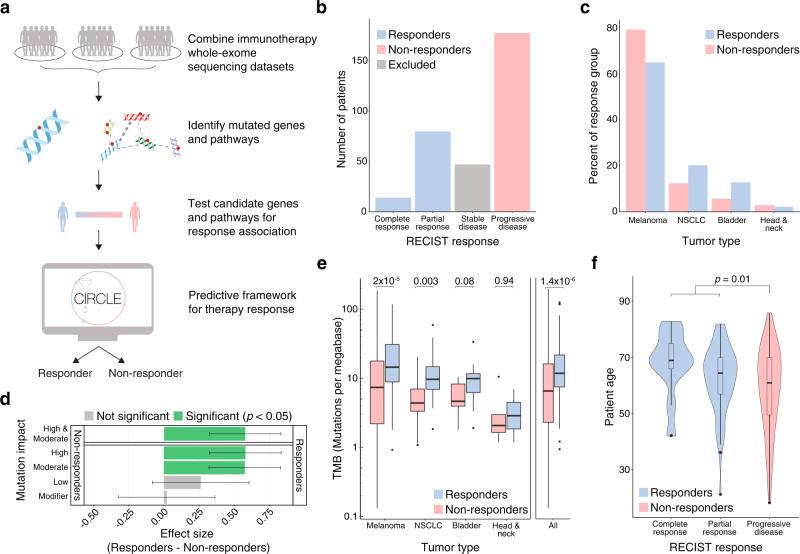
An aggregated cohort of immune checkpoint blockade (ICB) patients replicates known correlations between tumor mutational burden and age with treatment response. **a** Overview of the two-stage approach for immunotherapy response prediction. We pooled 6 cohorts of immune checkpoint blockade (ICB) recipients with matched whole-exome sequencing (WES) and Response Evaluation Criteria in Solid Tumors (RECIST) classification. We identified genes and pathways under positive selection and tested the nominated genes and pathways for their ability to predict ICB response. The significant predictors were used to develop and test an ICB response prediction algorithm. **b** Number of patients from the aggregated set of 6 cohorts in each RECIST response group. Patients with stable disease were excluded from analyses and the RECIST classifications of complete response and partial response were both considered responders. **c** Proportion of tumor types amongst ICB responders and non-responders. **d** Enrichment (effect size, Hedge’s *g*) for different types of mutations in responders (*n* = 94) and non-responders (*n* = 178) to ICB therapy. Error bars represent the 95% confidence interval and significance was determined using a two-sided Welch’s *t* test with Bonferroni correction. Tumor Mutational Burden (TMB) is the union of High and Moderate mutations. **e** TMB for responders (*n* = 94) and non-responders (*n* = 178) to ICB therapy by tumor type. Statistical significance was tested using two-tailed Welch’s *t* tests of log_2_ TMB. **f** Patient ages for different RECIST response groups (complete response *n* = 14, partial response *n* = 80, progressive disease *n* = 178). Statistical significance was tested using a two-tailed Welch’s *t* test. In **e** and **f**, the boxplot center line denotes median, with box limits being the 25th and 75th percentile. Boxplot whiskers indicate 1.5 times the interquartile range, while outliers above/below the whiskers are represented individually as points.

We aggregated WES and clinical (including RECIST categorization) data from six previously published immunotherapy studies^7,15–19^ encompassing 319 patients (Fig. 1a, Supplementary Table 1, 2). These studies included patients with diverse tumor types (melanoma, non-small cell lung cancer (NSCLC), bladder cancer, and head and neck cancer) with primarily pre-treatment WES and post-treatment RECIST categorization of ICB response. As expected, given the diverse tumor types, a large range of response rates was observed among the studies, ranging from 6 to 56% of patients with partial or complete response (Supplementary Table 2). Among these patients we identified; 14 complete responders, 80 partial responders, 47 patients with stable disease, and 178 with progressive disease. To study genomic predictors of ICB response, we dichotomized response data, treating complete and partial responders as “responders” and progressive disease patients as “non-responders” (Fig. 1b**)**. In total, these two groups contain 272 patients consisting of 202 patients with melanoma, 41 with NSCLC, 22 with bladder cancer, and 7 with head and neck cancer (Fig. 1c). Using this curated dataset, we sought to understand whether previously described correlates of ICB response were also predictive in our aggregated cohort.

To examine the correlation of TMB with ICB response, we categorized somatic mutations in  the tumors of responders and non-responders into four mutational impact classes (High, Moderate, Low, and Modifier) as defined by the SnpEff annotation and prediction framework^21^. Mutational burdens of High and Moderate impact mutations were found to be significantly different in responders when compared to non-responders (54.1 vs 36.7 mutations per patient respectively, Bonferroni-corrected Welch’s two-tailed *t* test of log_2_ (TMB), *p* = 2.6 × 10^−6^ for High, 534.8 vs 378.9 mutations per patient respectively, *p* = 1.5 × 10^−6^ for Moderate). Three studies^7,15,16^ were excluded from the analysis of Low and Modifier mutations (e.g. synonymous) as they reported few mutations of these classes (Supplementary Table 3). As expected, the burden of Low and Modifier mutations was not significantly different between responders and non-responders (Bonferroni-corrected Welch’s two-tailed *t* test of log_2_ (TMB), *p* = 0.07 for Low, *p* = 0.90 for Modifier) (Fig. 1d), despite their being present in tumor exomes at equal or greater abundance than High impact mutations (see Methods) (Supplementary Fig. 1a, b).

We further stratified TMB by variant classes, such as stop gain, missense, and synonymous, and found similar results (Supplementary Fig. 1c, d). For this study, we defined TMB as the sum of High and Moderate impact mutations, as these mutation classes capture non-synonymous mutations, reflecting the most commonly used definition of TMB^7,11,15–19,22–26^. We found that TMB was significantly higher in responders (1.4-fold more mutations in responders, Welch’s two-tailed *t* test difference of log_2_ (TMB), *p* = 1.4 × 10^−6^) (Fig. 1d). Other groups have also suggested that certain mutation types might be more predictive of immunotherapy response^11^.

We then stratified the analysis of TMB across the eligible tumor types and found significant associations with ICB response in melanoma and NSCLC (melanoma: 1.5-fold, Welch’s two-tailed *t* test *p* = 2.0 × 10^−5^; NSCLC: 2.5-fold, *p* = 0.003) and a positive trend amongst bladder and head and neck tumors (bladder: 1.9-fold, Welch’s two-tailed *t* test *p* = 0.08; head and neck: 1.1-fold, *p* = 0.94) (Fig. 1e). We also found a significant difference in age between ICB responders and non-responders (on average, responders were 4.5[0.9–8.0] years older, Welch’s two-tailed *t* test *p* = 0.01) and a significant positive correlation between age and the included RECIST response categories (Spearman’s rank correlation *r*_*s*_ = 0.14, *p* = 0.03) (Fig. 1f). In agreement with this result, Kugel et al.^4^ recently found that metastatic melanoma patients over the age of 60 had better responses to anti-PD1 checkpoint inhibitors than younger patients.

### Mutations in the transcriptional repressor gene *BCLAF1* are predictive of immunotherapy non-response

Previous genome-wide analyses of ICB response have primarily focused on global mutational patterns, under the premise that ICB responsive tumors will have a high burden of neoantigens^8,11^. However functional mutations at individual genes may alter tumor cells and make them more immunogenic or ICB resistant. For example, loss or mutation of *B2M* is an immune evasion mechanism that causes loss of class I MHC antigen presentation and may render tumors resistant to ICB therapy^27,28^. While most somatic mutations are neutral passengers, a subset of genes are under positive selection in tumors and frequently harbor functional mutations. Such genes can be identified through statistical approaches that model neutral mutational processes to identify genes that harbor an excess of mutations above background. To identify functional mutations that may mediate ICB response, we applied a two-stage biomarker discovery methodology: In the first “feature selection” phase, we identified positively selected genes in the cohort, irrespective of response data. In the second, “biomarker association phase”, we assessed the features nominated in the first phase for their correlation with immunotherapy response in a multivariate logistic model.

**Fig. 2:**
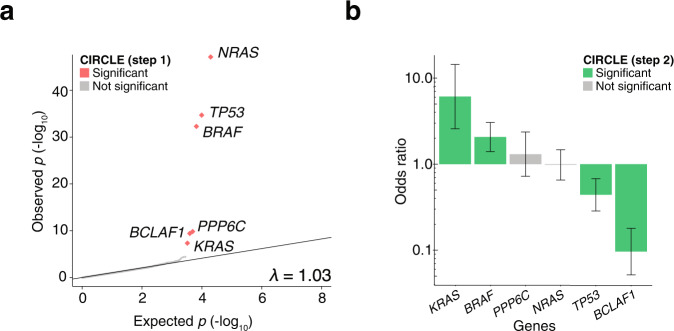
A two-stage approach identifies *BCLAF1* somatic genotype  as a predictor of ICB response. **a** Quantile-quantile plot of fishHook *p*-values to assess significance of gene mutational burden after removing confounders. The *p*-values were obtained by comparing observed mutational rate to the right tail (one-sided) of the expected mutational rates derived from a gamma-Poisson model of genome-wide mutational density and the covariates replication timing, epigenetic state, and sequence context. In the first stage of CIRCLE, six significant genes were identified below a false-discovery threshold (FDR < 0.1). **b** Odds ratios (ORs) of response to ICB therapy in patients with a high or moderate impact mutation in the indicated gene as compared to patients that do not have a high or moderate mutation in the given gene (*n* = 272 patients). Error bars indicate the 95% confidence interval of the odds ratio. ORs greater than one indicate enrichment in responders and ORs less than one indicate enrichment in non-responders. Statistical significance was tested using a two-sided Wald’s test of coefficients with multiple-hypothesis correction (FDR < 0.2).

To identify positively selected genes and pathways in the aggregated immunotherapy cohort, we adapted fishHook, a statistical method originally developed to study noncoding mutational recurrence in whole genome sequencing^20^. We limited the fishHook analysis to the coding regions of 19,688 genes that are consistently captured by WES. To nominate genes under positive selection, we corrected for several known determinants of neutral genome-wide mutational density, including replication timing, sequence context, and chromatin state^29^. In total, we examined 129,344 High and Moderate impact mutations from our cohort, excluding any mutations occurring at bases that were covered in less than 80% of patients from The Cancer Genome Atlas (TCGA) WES datasets. From this, we identified six recurrently mutated genes using a significance threshold of *q* < 0.1: *BCLAF1*, *BRAF*, *KRAS*, *NRAS*, *PPP6C*, and *TP53*. Using a quantile-quantile plot (Fig. 2a), we observed a genomic *p*-value inflation factor (λ) of 1.03, indicating adequate modeling of neutral mutational processes.

The somatic genotypes of six genes were then tested for their ability to predict response using logistic regression with Age, tumor type, log_2_ (TMB), and Study of Origin as covariates. Of these genes, four (*BCLAF1, KRAS, BRAF,* and *TP53*) were significantly predictive following multiple-hypothesis correction (*q* < 0.2). The top hit, *BCLAF1* (BCL2 Associated transcription Factor 1), was depleted in responders (odds ratio of mutation status in responders to non-responders = 0.096 [0.026–0.304], Wald’s test of coefficients *p* = 0.0002, *q* = 0.001) (Fig. 2b).

*BCLAF1* encodes a transcriptional repressor that regulates the type 1 interferon response^30^. Knockdown of BCLAF1 led to decreased STAT1 and STAT2 phosphorylation, and increased susceptibility to infection by alphaherpesvirus in lung and brain tissue of mice. BCLAF1 also interacts with STAT2 and interferon-stimulated response elements to enhance the transcription of interferon response genes. *BCLAF1*-null T-cells have impaired development and do not respond to TCR and CD28 stimulation even in the presence of IL-2^31^. BCLAF1 has also been shown to function downstream of NF-KB to upregulate IL-8^32^, is regulated by SIRT1^33^, and plays a role in DNA damage response^34^.

**Fig. 3:**
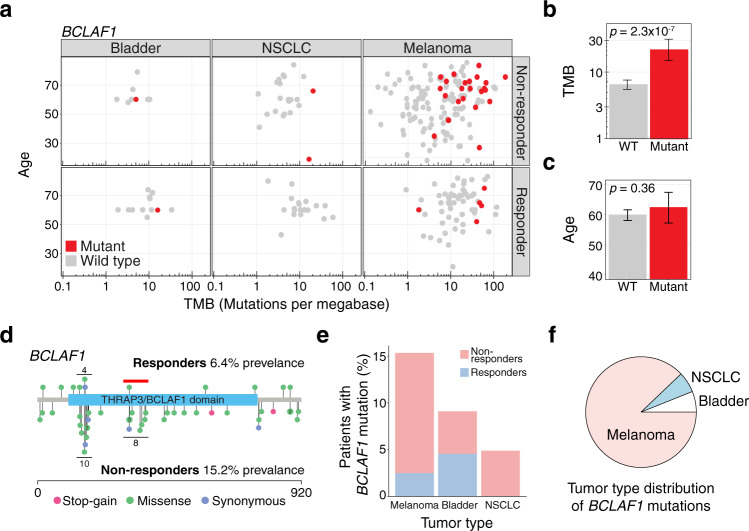
*BCLAF1* mutations identify a subset of non-responders with high tumor mutational burden (TMB). **a** Age, TMB and tumor type for responders and non-responders with (*red*) and without (*gray*) *BCLAF1* mutations. **b** TMB of patients with (*n* = 33) and without (*n* = 239) mutations in *BCLAF1*. Significance was calculated using a two-sided Welch’s *t* test and error bars indicate 95% confidence intervals. **c** Age of patients with (*n* = 33) and without (*n* = 239) mutations in *BCLAF1*. Significance was calculated using a two-sided Welch’s *t* test and error bars indicate 95% confidence intervals. **d** Protein location of mutations in BCLAF1 in responders (*top*) and non-responders (*bottom*). Mutations are color-coded by mutation type. Horizontal black lines indicate mutational clusters. The red horizontal line indicates a mutational cluster not present in responders. **e** Prevalence of *BCLAF1* mutations in melanoma, bladder, and NSCLC cancer by ICB response status. **f** Distribution of *BCLAF1* mutations by tumor type.

*BCLAF1* mutations were present in 15.2% of non-responders and only 6.4% of responders (Fig. 3a**)**. Furthermore, *BCLAF1* mutations were enriched in older melanoma patients with high TMB: When testing for this association, we found that patients with *BCLAF1* mutations had higher log_2_ (TMB) (9.4) than *BCLAF1* WTs (7.6, Welch’s two-tailed *t* test, *p* = 2.3 × 10^−7^) (Fig. 3b**)**, but that there was no significant difference in age between *BCLAF1* mutants (62 years) and WTs (60 years, Welch’s two-tailed *t* test, *p* = 0.36) (Fig. 3c**)**. Given these results, we divided patients into a TMB-high group (>10 mutations/megabase)^35,36^ and a TMB low group (<10 mutations/megabase)^35,36^, and observed that *BCLAF1* was significantly associated with response in the TMB-high group (OR = 0.25 [0.07–0.78], Fisher’s exact *p* = 0.01), but not in the TMB low group (OR = 0.33 [0.01–2.60], Fisher’s exact *p* = 0.44). These results suggest that *BCLAF1* mutations may identify a unique subset of TMB-high non-responders.

To better understand the functional context of *BCLAF1* mutations, we plotted each mutation across the BCLAF1 protein sequence, separated by response status. We identified two mutation clusters within a Pfam functional domain (PF15440, THRAP3/BCLAF1 family), one of which was present only in non-responders (Fig. 3d). *BCLAF1* mutations were present across multiple tumor types; melanomas had the highest overall prevalence (14.4%), with bladder cancer (9.1%) and NSCLC (4.9%) also harboring *BCLAF1* mutations (Fig. 3e, f). Given these differences and the wide range of response rates among the various studies and tumor types in our dataset, we explicitly tested if *BCLAF1* mutations acted as a surrogate for tumor type or study of origin. We found no significant difference in the frequency of *BCLAF1* mutations across the various tumor types and studies of origin when compared to the overall frequency of *BCLAF1* mutations (two-tailed Fisher’s exact test, *p* > 0.05 for all tumor types and studies of origin).

Among the other predictive genes (Supplementary Fig. 2), *BRAF* and *KRAS* mutations were enriched in responders (*BRAF*: OR = 2.1, *q* = 0.09; *KRAS:* OR = 6.1, *q* = 0.09), while *TP53* mutations were enriched in non-responders (OR = 0.44, *q* = 0.09). In our aggregated cohort, the tumor type distributions among *BRAF*, *KRAS*, and *TP53* were as expected, with *BRAF* exhibiting a strong bias towards melanoma, *KRAS* exhibiting a strong bias towards NSCLC and *TP53* exhibiting a pan tumor-type distribution (Supplementary Fig. 3). In total, we identified 4 ICB response predictive genes from our logistic regression (*BCLAF1*, *BRAF*, *KRAS*, and *TP53*).

### MAPK-ERK pathways are biomarkers of ICB response

Since certain cancer genes (e.g. *BRAF*) share pathways with other more rarely mutated targets of driver alteration (e.g. *ARAF*, *RAF1*), it may be useful to consider mutational status in a set of genes as a predictive biomarker. To expand our two-stage biomarker discovery approach to multi-gene biomarkers, we applied fishHook^20^ to a collection of gene sets from the Reactome database (*n* = 2022 pathways)^37^. We nominated 199 recurrently mutated pathways (Supplementary Fig. 4a) across the 272 profiled cases (*q* < 0.1) in the first feature selection stage.

**Fig. 4:**
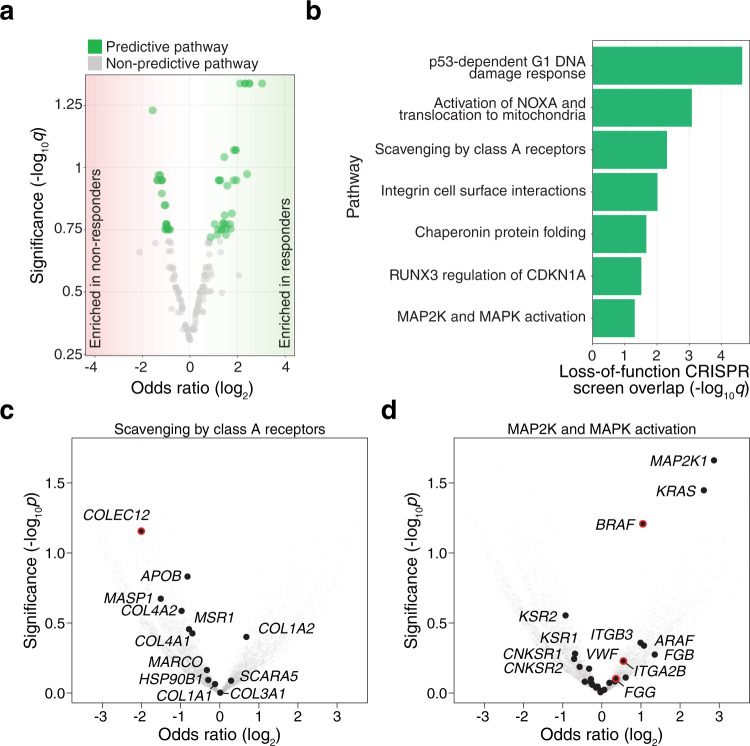
Somatic mutations in genes encoding DNA damage, immune-associated, and mitogen-activated protein kinase (MAPK) pathways correlate with ICB response. **a** Volcano plot of fishHook-nominated pathways with log_2_ odds ratio for ICB response (*x*-axis) and significance of association with ICB response (*y*-axis). Statistical significance was tested using a two-sided Wald’s test of coefficients with multiple-hypothesis correction (FDR < 0.2). **b** fishHook-nominated pathways that overlap top-ranked genes from a genome-wide CRISPR screen for immunotherapy resistance (FDR-corrected one-sided hypergeometric test). **c**–**d** Volcano plots of odds ratio (responders/non-responders) and nominal *p*-values for genes in two of the fishHook-nominated pathways: Scavenging by Class A Receptors (**c**) and MAP2K and MAPK Activation (**d**). Red outlines indicate genes that were also found in the CRISPR screen. Indicated *p*-values are from the fishHook model using the observed mutation rate for each gene.

In the second stage, we correlated pathway mutational status with ICB response using Age, tumor type, TMB and Study of Origin as covariates in a logistic regression model similar to our gene level analysis (see above). After multiple-hypothesis correction, 54 pathways were found to be significant predictors of response (*q* < 0.2) **(**Fig. 4a, Supplementary Table 4**)**. To minimize the redundancy of pathways with many shared genes, we ordered the nominated pathways by significance and excluded pathways that shared greater than 40% of genes (see Methods) with more significant pathways **(**Supplementary Fig. 4b, Supplementary Table 5).

Of the 21 remaining pathways, we conducted further gene level analysis and found that 9 of the 21 pathways contain *TP53*, 7 pathways contained either *BRAF* or *KRAS*, and that there was no overlap between the *TP53*-containing pathways and the *BRAF*/*KRAS*-containing pathways. Five pathways did not contain any of the previously identified genes: “Integrin cell surface interactions” (*q* = 0.12, OR = 3.59 [1.25–11.18]), “Assembly of collagen fibrils and other multimeric structures” (*q* = 0.17, OR = 2.96 [0.99–9.79]), “CD28 dependent Vav1 pathway” (*q* = 0.17, OR = 0.47 [0.20–1.03]), “FMO oxidizes nucleophiles” (*q* = 0.18, OR = 2.11 [0.90–5.03]), “Scavenging by Class A Receptors” (*q* = 0.18, OR = 0.48 [0.20–1.08]).

To understand the functional implications of mutations in these genes, we compared the genes in these pathways with our recent genome-wide pooled CRISPR screen for immune evasion^38^. This forward genetic screen targeted virtually all genes (*n* = 19,050 genes and 1864 microRNAs) in human melanoma to identify loss-of-function mutations that drive resistance to adoptive T-cell immunotherapy. Specifically, we examined the overlap between WES-derived pathway predictors and the enriched candidate genes from this functional genomic screen. The enriched genes in this CRISPR screen significantly (*q* < 0.1) overlapped with 7 of the 21 pathways (see “Methods”) (Fig. 4b, Supplementary Table 6). To further explore the overlapping pathways at the gene level, we tested each gene within a given pathway using the same logistic regression method as in the gene-level analysis and plotted the log_2_ (odds ratio) (responder/non-responder) and the nominal *p*-value for each gene. Several pathways exhibited multi-gene trends towards either responders or non-responders (Fig. 4c, d, Supplementary Fig. 4c–g).

Four of the 7 overlapping pathways contained *TP53*. p53-Dependent G1 DNA Damage Response, which had the most significant overlap with the CRISPR screens, including *UBA52*, *CCNE1*, and eight genes that encode proteasome subunits (*PSMB5*, *PSMA6*, *PSMC2*, *PSMD7*, *PSMA5*, *PSMB2*, *PSMA7*, *PSMB4*). Activation of NOXA and Translocation to Mitochondria overlapped with two CRISPR screen genes (*PMAIP1*, *E2F1*). Chaperonin-mediated Protein Folding which overlapped with seven CRISPR screen genes (*GNB3*, *CSNK2B*, *TUBA1C*, *GNAT2*, *CCNE1*, *NOP56*, *TUBB2B*), and RUNX3 Regulation of CDKN1A Transcription which overlapped with one CRISPR screen gene (*ZFHX3*). One pathway (MAP2K and MAPK Activation) contained *BRAF* and overlapped with three CRISPR screen genes (*BRAF*, *ITGA2B*, *FGG*). The last two pathways (Scavenging by Class A Receptors and Integrin Cell Surface Interactions) did not contain genes identified in the gene-level analysis. Scavenging by Class A Receptors contained three CRISPR screen genes, *COLEC12* and *APOE* which are both associated with Alzheimer’s Disease^39,40^, and *CALR* which encodes a chaperone for MHCI folding^41^. Integrin Cell Surface Interactions overlapped with six CRISPR screen genes; ICAM1 which functions in leukocyte adhesion^42^, VTN which functions in macrophage adhesion^43,44^, two integrin subunits (ITGA2B, ITGB1), a collagen subunit (COL18A1), and the gamma component of fibrin (FGG) (Supplementary Table 6).

### Combining identified genes and pathways is superior to tumor mutational burden alone for predicting patient response to checkpoint blockade

Next, we sought to quantify whether somatic mutations in the genes and pathways that we identified could improve our ability to predict immunotherapy response over TMB alone. We combined the significantly predictive genes (*BCLAF1, TP53, KRAS*, and *BRAF*), the predictive pathways that overlapped with prior functional genomic screens (p53-Dependent G1 DNA Damage Response, Activation of NOXA and translocation to mitochondria, Chaperonin mediated protein folding, RUNX3 regulates CDKN1A transcription, MAP2K and MAPK activation, Scavenging by Class A Receptors, and Integrin cell surface interactions), and baseline features (age, TMB, and tumor type) into a multivariate logistic predictor of immunotherapy response. We term this predictive framework the Cancer Immunotherapy Response CLassifiEr (CIRCLE). To build CIRCLE, we fit a logistic regression model based on these features to the ICB response data and tested its ability to predict immunotherapy treatment response (Supplementary Table 7).

**Fig. 5:**
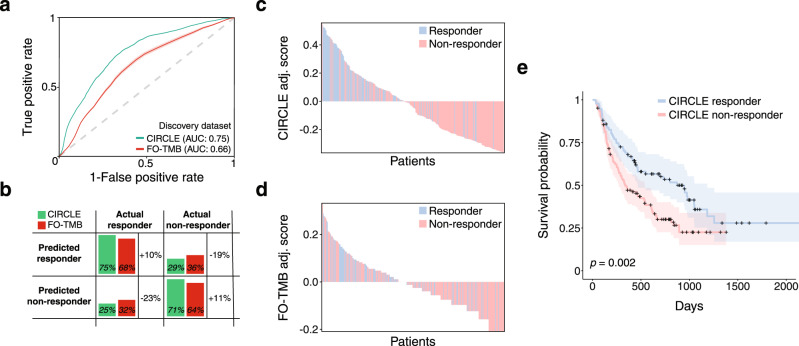
The cancer immunotherapy response CLassifiEr (CIRCLE) predicts ICB response and patient survival. **a** Averaged areas under the receiver-operator curve (AUCs) from 100 Monte Carlo cross validation iterations of the CIRCLE classifier and the FoundationOne CDx tumor mutational burden (FO-TMB) classifier. Error shading indicates the standard deviation of AUCs calculated from the 100 cross validation iterations. **b** Absolute values and percent change in the true positive rate (sensitivity), true negative rate (specificity), false positive rate and false negative rate of the CIRCLE classifier and FO-TMB classifier. **c**–**d** Waterfall plots of per patient scores from the CIRCLE (**c**) and FO-TMB (**d)** classifiers. Each patient is represented as a vertical bar and the adjusted score is equal to the score of the indicated classifier minus the optimal cutoff derived from the respective receiver operating characteristic curves. **e** Kaplan–Meier plot of overall survival for patients classified as CIRCLE responders versus CIRCLE non-responders. Shaded areas indicate the 95% confidence interval. Statistical significance was calculated using a two-sided Cox proportional hazards test with tumor type as a covariate.

To benchmark CIRCLE, we compared it against a simulated version of FoundationOne CDx (FO), a clinically-available, FDA-approved companion diagnostic that reports mutations found in a preselected set of genes^8,45,46^. FO estimates TMB by counting non-synonymous and protein-coding mutations across a panel of 323 genes^47^. To simulate the FO diagnostic, we filtered the WES data for these 323 genes and computed TMB (“FO-TMB”). We then built a logistic regression classifier by fitting the FO-TMB to ICB response data. Using cross-validation, we found that CIRCLE resulted in better prediction than FO-TMB as calculated by the area under the receiver operating characteristic curve (AUC) (mean CIRCLE AUC: 0.75 95% CI [0.74–0.76], mean FO-TMB: 0.66 95% CI [0.65–0.67]) (Fig. 5a). We also calculated the AUCs for the consensus of the cross-validation classifications and found a similar difference in AUC between CIRCLE (AUC: 0.73) and FO-TMB (AUC: 0.63) (DeLong *p* = 0.006)^48^.

We also computed the sensitivity (true positive rate), specificity (true negative rate), and harmonic mean of precision and recall (F1-score) of CIRCLE and FO-TMB. When using CIRCLE, we found a 10.5% increase in sensitivity (CIRCLE: 75.5%, FO-TMB: 68.3%), a 11.0% increase in specificity (CIRCLE: 70.9%, FO-TMB: 63.8%) (Fig. 5b), and a 14% increase in the F1-score (CIRCLE: 0.65, FO-TMB: 0.57).

To better understand the improved prediction, we tested each of the following subsets of the CIRCLE feature set for their predictive ability: baseline features (age, TMB, and tumor type; consensus AUC: 0.65), genes (AUC: 0.56), and pathways (AUC: 0.62) (Supplementary Fig. 5a–d). Baseline features and genes together yielded an AUC of 0.69 (compared to baseline features alone: DeLong *p* = 0.11); however, baseline, genes, and pathways together yields an AUC of 0.73 (compared to baseline features alone: *p* = 0.02; compared to baseline features and genes: *p* = 0.16) (Supplementary Fig. 5e). Importantly, pathways were not redundant with genes: Genes and pathways together had an AUC of 0.69 (compared to genes alone: *p* = 4 × 10^−4^; compared to pathways alone: *p* = 4 × 10^−3^).

CIRCLE scores (the probability of response under the logistic regression model) also yielded a better separation of responders and non-responders than FO-TMB scores in aggregate (standardized difference of mean predictive scores in responders and non-responders: Θ = 1.10 for CIRCLE, 0.51 for FO-TMB) (Supplementary Fig. 5f, g) and on an individual patient level (Fisher’s exact test for association between classifier assigned and true response status, CIRCLE: OR = 9.9 95% CI [5.33–19.11], *p* < 2.2 × 10^−16^; FO-TMB: OR = 3.04 95% CI [1.76–5.29], *p* = 3.1 × 10^−5^) (Fig. 5c, d). We also examined the precision versus recall curves of the CIRCLE model as compared to FO-TMB and observed an area under the precision recall curve (AUPRC) of 0.57 for CIRCLE and 0.45 for FO-TMB (Supplementary Fig. 5h–l).

Although CIRCLE was trained to predict ICB response, we asked whether CIRCLE scores were also correlated with overall survival (OS). For this purpose, we stratified patients based on their CIRCLE response classification and found that CIRCLE responders also had increased OS (two-sided Cox proportional hazards with tumor type as a covariate: *p* = 2 × 10^−3^, comparing CIRCLE classification to OS, *p* = 10^−4^ comparing CIRCLE score to OS) (Fig. 5e). A natural question is whether CIRCLE is truly predictive or whether biomarkers that correlate with therapy response may just be indicative of a milder disease subtype. For this purpose, we examined 2184 patients from the TCGA PanCancer Atlas cohort^49^ whose tumor types were present in our cohort: non-small cell lung cancer (*n* = 368 for squamous cell carcinoma and *n* = 467 for adenocarcinoma), melanoma (*n* = 361 cutaneous and *n* = 80 uveal), head and neck squamous cell cancer (*n* = 514), and bladder cancer (*n* = 394). Using each patient’s clinical features and WES data, we computed CIRCLE scores. Within the TCGA PanCanAtlas cohort, we observed that CIRCLE responders and non-responders had comparable OS in the whole cohort and the individual tumor types (Supplementary Fig. 6), supporting our conclusion  that the CIRCLE score is predictive and not merely prognostic.

Finally, we tested the generalizability of the CIRCLE model with independent validation cohorts not used in the development or training of our model. We selected one melanoma cohort^22^ (*n* = 124) and one non-small cell lung cancer cohort^23^ (*n* = 41). In these independent validation cohorts, the CIRCLE classifier had an AUC of 0.61 (OR = 2.73, Fisher’s exact *p* = 0.003) (Supplementary Fig. 7a). The AUC for TMB was also 0.61. To determine how much additional predictive ability CIRCLE provides beyond TMB, we fit a logistic regression model for true response with CIRCLE prediction and TMB-high status (>10 mutations per Mb) as independent variables^35,36^. We observed that CIRCLE scores yielded a significant increase in prediction over a model with TMB alone (*p* = 0.02, two-tailed Wald’s test). *BCLAF1* showed a non-significant trend for enrichment in TMB-high non-responders (OR = 0.67 [0.19–2.33], *p* > 0.05) which emphasizes the need for additional pan-cancer data to determine if the *BCLAF1* association generalizes widely. We also found improved survival among CIRCLE responders (CIRCLE score *p* = 0.022, CIRCLE responder/non-responder *p* = 0.011) in the subset of validation cohort cases (*n* = 124) with reported OS data (Supplementary Fig. 7b). While CIRCLE and TMB yielded the same AUCs for response prediction in the validation cohort, joint analysis of these features in a logistic regression model showed that the CIRCLE score was independently predictive of response above TMB. Taken together, these results support broader investigations into CIRCLE and more generally recurrent somatic alterations as immunotherapy biomarkers.

## Discussion

Previous studies have used a variety of different biomarkers to predict ICB response, including tumor mutations found in candidate genes^27,50^, mutations found through wholegenome sequencing or WES^7,15–19^, transcriptomics^12,13^, tumor mutational burden^8,51^, T cell diversity/clonality^52,53^, and neoantigen production^54,55^. In addition, recent work has integrated multiple different biomarkers, such as combining tumor mutational burden, DNA sequencing, and RNA sequencing^50^. Our study focuses on biomarkers derived from existing cohorts of immunotherapy patients with paired WES and response data alongside clinically relevant metadata. It capitalizes on the advantages of both candidate gene and genome-wide approaches to achieve optimized predictive power with a modest cohort size. Using several previously published studies, we assembled a larger cohort of WES profiled tumors  than in many recent studies^7,15–19^. Then, via analysis of positive somatic selection, we nominated a small set of genes and pathways enriched in likely functional mutations. Mutation status in these genes and pathways enabled superior prediction of cancer ICB response when compared to previously reported metrics such as TMB.

Our results add to a growing body of evidence implicating *KRAS* mutations in immunotherapy resistance. Recently, Van Allen and colleagues also noted that *KRAS* mutations correlate with ICB response in a WES meta-analysis (partially overlapping with our study)^19^; however, *KRAS* mutations were nominally but not genome-wide significant in that analysis. A separate targeted sequencing study in 47 NSCLC patients treated with anti-PD1 inhibitors found that patients with *KRAS* mutant tumors have a longer progression-free survival (PFS) and overall survival (OS) than *KRAS* wild-type patients (hazard ratio [HR] = 0.48, *p*  =  0.04)^56^. Other groups have demonstrated that *KRAS* mutation status in NSCLC is associated with an inflammatory tumor microenvironment, including PD-L1 expression and CD8+ tumor-infiltrating lymphocytes^57^. But this result may be specific to lung cancer, as others have shown that in colorectal cancers, mutant *KRAS* can repress interferon response genes^58^. As our meta-analysis cohort did not include colorectal cancers, we are unable to discern the role of *KRAS* mutations in treatment response for these cancers. A study in 52 patients with NSCLC also found that patients with *TP53* mutations had a higher risk of progression regardless of PD-L1 expression (HR = 3.3), although the result was not significant (*p* = 0.05)^59^. Our correlation between *BRAF* mutations and ICB response is discordant with data from recent trials showing similar responses and durability of responses in patients with *BRAF* wild-type and *BRAF* mutant melanoma^60^.

We found that the CIRCLE classifier yields improved ICB response prediction when compared to TMB. Larger immunotherapy cohorts will be needed to validate this finding, and more broadly the principle that positively selected driver alterations can help predict immunotherapy response. Larger pan-cancer cohorts will allow us to test the assertion that *BCLAF1* helps identify TMB-high ICB non-responders. Due to the cancer type specificity of driver alterations, we can expect that expanding CIRCLE to broader pan-cancer cohorts will require the classifier to be revised with additional discovery analyses. Such analyses will employ the two-stage approach to nominate additional tumor-type relevant genes and pathways and correlate their somatic genotypes with immunotherapy response, similar to the approach taken in our study. We foresee that such expanded CIRCLE classifiers will provide valuable information that may in the future help guide treatment choice in the clinic, particularly as the scope of immunotherapy broadens to additional cancer types.

With the extraordinary cost and the serious side effects associated with ICB, there is a major unmet need for response biomarkers. While panel testing is already used routinely in immuno-oncology, our results suggest that the use of broader diagnostics (including WES and whole genome sequencing) may significantly improve this stratification of responders and non-responders. A key practical challenge in clinical implementation of the CIRCLE classifier is the need for WES to assess mutation status at genes and pathways that are not commonly included on cancer gene panels (e.g. *BCLAF1)*. Aside from the formidable issues of cost and logistics, one obstacle to routine whole exome or genome sequencing is the perception that genes which are not currently assayed by clinical gene panels have limited current or near-term clinical relevance. In full awareness of this perception, we hope that our study and other similar analyses will motivate more formal and prospective explorations into the routine clinical utility of these broader genomic assays.

## Methods

### Data curation

We aggregated WES data from immunotherapy patients with matched Response Evaluation Criteria in Solid Tumors (RECIST) classification from six previously published studies^7,15–19^. We analyzed 319 patients, labeling 94 patients with Partial Response (PR) or Complete Response (CR) as Responders and 178 patients with Progressive Disease (PD) as Non-Responders. We term this group of 272 patients the immunotherapy cohort. Model cross-validation was conducted using randomly assigned train-test splits of 75% test (*n* = 204) and 25% train (*n* = 68). Survival analysis was conducted using the subset of patients with survival data (*n* = 253). Due to the different end points of the studies, all of the patients were right censored.

Data was aggregated such that the following fields were retained; Original Study ID, RECIST Classification, Sex, Age, TNM Staging, Survival, Vital Status (at end of follow-up), tumor type, Treatment Drug, and Stage. To minimize over-stratification, we combined all variants of melanoma (e.g. uveal, skin) into a single ‘melanoma’ category. Additional annotation of the data included SnpEff variant classification^21^ for each mutation within the dataset. SnpEff was primarily used to predict the functional impact of mutations as “High”, “Moderate”, “Low” or “Modifier”. Patients without age metadata were assigned an age equal to the mean of the age for all patients with age metadata.

### Biomarker analysis

#### TMB and age analyses

We analyzed TMB in responders and non-responders using two-tailed Welch’s *t* tests with log_2_ of TMB to achieve more normally distributed values. TMB was defined as the total number of “High” and “Moderate” SnpEff mutations present within a patient’s WES data. High and Moderate mutations include the following subclasses: missense variants, variants that impact protein-protein contact, splice acceptor variants, splice donor variants, start lost variants, stop gained variants and stop lost variants^21^. As a control, we tested whether Low and Modifier mutations might be underrepresented, thus making it more challenging to detect significance. To this end, we tested for significant differences between High, Moderate, Low, and Modifier mutations using a one-way ANOVA (*p* < 2 × 10^−16^). We find that Modifier mutations do not occur at a significantly different frequency than High impact mutations (post hoc Welch’s two-sample *t* test, *p* = 0.71), and that Low impact mutations occur at a higher frequency than High impact mutations (post hoc Welch’s *t* test, *p* < 2 × 10^−16^).

FoundationOne TMB is calculated as the total number of “Moderate” and “High” mutations that fell within genes that are included as part of the FoundationOne panel^8^, although this results in a similar TMB-based prediction of response. That is, there is no significant difference between response prediction based on TMB calculated from WES and response prediction based on the simulated FoundationOne Panel TMB (AUC = 0.67 for exome TMB and 0.66 for panel TMB, DeLong *p* = 0.25). The effect size for TMB was calculated as the Hedge’s *g* statistic, the difference of means of log_2_ (count) of a given mutation class divided by an estimated combined standard deviation weighted by sample sizes, using the esc R package.

We analyzed age in responders and non-responders using a two-tailed Welch’s *t* test of age and a Spearman’s rank correlation test, where ranking of included RECIST categories proceeded as: Progressive Disease, Partial Response, Complete Response.

#### Gene nomination

The first step of the two-step biomarker nomination was performed by adapting the fishHook R package (https://github.com/mskilab/fishHook)^20^ to identify recurrently mutated genes across the coding subset of the WES mutation data. Briefly, fishHook fits a gamma-Poisson model to estimate expected neutral mutational counts from mutation data while correcting for linear covariates, such as replication timing, chromatin state, and sequence context. It then compares the observed mutational rates to the estimated neutral model to assess significance. This method was previously used to identify noncoding regions that were recurrently mutated in the wholegenome sequences of human cancers^20^. The specification of a fishHook model requires a set of mutations, a set of hypotheses, an eligible subset of the genome, and zero or more genomic (numeric or interval) covariates. each defined as genomic intervals. Covariates represent sequence-derived (e.g. GC content) or cell type-specific features (e.g. chromatin state, replication timing) that drive regional differences in neutral mutation density. The method then compares the observed and expected density of mutations among the eligible bases of hypotheses after applying a background linear model that uses the average value of each covariate across eligible bases of each hypothesis as a predictor.

To adapt fishHook to the analysis of protein-coding genomic regions consistently captured in WES experiments, we explored 19,688 GENCODE genes (build 19) that also had metadata on GeneCards^61^. We then defined the eligible subset as coding sequences (CDS) in which >80% of TCGA patients had sequencing coverage. For mutations, we used SNVs and indels that SnpEff classified as ‘Moderate’ or ‘High’ impact (*n* = 129,344 mutations). Given the multi-tumor type dataset (spanning melanoma, bladder cancer, NSCLC and head and neck cancer), we developed a custom pan-cancer covariate set (“covariome”) to comprehensively capture the contribution of background genomic features to the neutral mutation density. Briefly, we defined three types of biological covariates; replication timing across 96 cell lines, 15 ChromHMM states across 127 cell lines and tissues, and sequence context (mono, di and tri). Replication timing and epigenomic data were obtained from the ENCODE and Roadmap Epigenomics **(**Supplementary Data 1) projects, respectively^62,63^. Sequence context was derived from the hg19 human reference genome. This yielded 96, 1905, and 98 replication, chromatin, and sequence context-driven covariates, respectively. To reduce the dimensionality of the fishHook analysis we used the first 50, 200, and 50 principal components (PCs) of replication timing, ChromHMM states, and sequence context respectively, yielding a final covariate set of 300 PC derived numeric covariates.

We extended the model to enable the nomination of pathways under somatic selection. Briefly, given a fishHook model fit across *n* genes yielding an expected mutation count ${e}_{i}$ at gene *i*, $,i\in 1,\ldots ,n$, we then assessed the significance of gene set $I\subset 1,\ldots ,n$ by fitting the gamma-Poisson regression *y*_*i*_ ~ offset (log *e*_*i*_) and taking the magnitude and *p-*value of the fitted intercept as the pathway-level effect size and significance.

In total we tested 19,688 genes and 2022 Reactome pathways^37^, with a maximum gene/pathway contribution per patient equal to 1 mutation. Genes were nominated using a *q* < 0.1 threshold where *q*-values were calculated using the Storey method^64^.

#### Pathway nomination

In addition to looking for recurrently mutated genes, we organized sets of genes into pathways and used fishHook to nominate recurrently mutated pathways using identical parameters to the gene level analysis. Using this approach, we initially nominated 199 pathways as recurrently mutated. A high genomic inflation factor λ (slope linking observed -log_10_ *P* values to their expected quantiles) was observed (*λ* = 6.52), and we hypothesized that this was due to the repetition of the recurrently mutated genes among partially overlapping pathways. Upon removal of all pathways containing any of the 7 previously nominated genes, a λ of 1.17 was observed (Supplementary Fig. 4a). In total, 162 of the 199 nominated pathways contained one of the 7 previously nominated genes. We continued the analysis using the full set of 199 nominated pathways, as we wanted to make sure that we did not miss any associations between ICB response and pathways containing key cancer genes such as *TP53*, *BRAF*, and *KRAS*, all of which were among the 7 previously nominated genes. Pathways were nominated using a *q* < 0.1 threshold where *q*-values were calculated using the Storey method^64^. We calculated the significance of overlap between the CRISPR screen nominated genes and the immunotherapy cohort nominated genes using a hypergeometric test.

#### ICB response prediction

Biomarker nomination of genes and pathways was conducted using a two-tailed Wald’s tests of logistic regression coefficients. Each fishHook-nominated gene/pathway was converted to a binary feature such that 1 indicated that the patient had either a High or Moderate impact mutation anywhere in the given gene or in the case of pathways any High or Moderate impact mutation within any gene in the pathway. 0 indicated that the patient did not have such a mutation in the given gene/pathway. The association between the binary response variable and the gene/pathway feature was modeled as:

$${{{{{\rm{Response}}}}}} \sim\,	 {{{{{\rm{Logistic}}}}}}({\alpha }_{0}+{\alpha }_{1}{{{{{\rm{HasMutation}}}}}}+{\alpha }_{2}{{{{{\rm{TumorType}}}}}}+{\alpha }_{3}{{{{{\rm{Age}}}}}}\\ 	+{\alpha }_{4}{\log}_{2}({{{{{\rm{TMB}}}}}})+{\alpha }_{5}{{{{{\rm{StudyofOrigin}}}}}})$$

with Age, log_2_ (TMB), Study of Origin and tumor type as covariates (all previously identified biomarkers of ICB response). Multiple-hypothesis testing for genes and pathways utilized Storey *q*-values^65^ with a significance threshold of *q* < 0.2 (λ = 0).

The odds ratios for each tested genomic biomarker were calculated as *e*^*α*^ where *α* is the fitted coefficient of the logistic regression model. Confidence intervals were similarly calculated based on the confidence intervals of the coefficient. Mutation plots were constructed using the lollipops R package^66^ where each reference-alternate amino acid pair was plotted as a unique mutation.

### Model validation

We fit a logistic regression model of selected genes, pathways, tumor type, log_2_ (TMB), and Age to immunotherapy response, and named this model the Cancer Immunotherapy Response CLassifiEr (CIRCLE). We created a similar logistic classifier for comparison using a simulated FO-TMB measurement where we counted the number of High and Moderate impact mutations across the FO panel of 323 genes^47^. We computed specificity, sensitivity, AUROC and F1 scores for CIRCLE and FO-TMB classifiers using the means of 100 Monte Carlo cross-validation iterations of training (75%) and testing (25%) splits from the immunotherapy cohort. An aggregate ROC curve was derived by averaging the ROC curve from each iteration. The proportion of tumor types was kept constant between the testing and training sets for each iteration and across iterations. For each cross-validation iteration, we calculated the optimal cutoff (closest to point (0,1)) from the averaged ROCs and used it to assign scores and response classifications to each patient. Patients with CIRCLE or FO-TMB classifier scores greater than their associated cutoffs were classified as CIRCLE/FO-TMB responders respectively. DeLong *p*-values were calculated by first having the classifiers for CIRCLE and FO-TMB from each of the 100 iterations vote by a simple majority on the classification of each patient. We then used the pROC R package to implement the DeLong comparison method of AUCs^48^. We also performed 10-fold cross-validation (not Monte Carlo) and found that the mean AUCs were not significantly different (Monte Carlo CV: 0.752, 10-fold CV: 0.746, DeLong *p* = 0.11).

Survival analysis was conducted using the survival and survminer R packages, comparing the CIRCLE patient response classifications using a log-rank test or two-sided Cox proportional hazards model. For validation of ICB cohorts, tumor type was used as a covariate for Cox regression, while for the TCGA cohort, tumor type, age, stage, and *TP53* mutational status were used as covariates. Survival curves used the Kaplan-Meier estimator and were performed using the survival package.

### Additional software packages

Each studies’ data was downloaded from their associated publication and combined in R version 3.4.3^67^. All subsequent analysis was conducted in R 4.0.2^67^ and the following packages were used: abind 1.4-5, bayestestR 0.10.0, BiocGenerics 0.34.0, broom 0.7.6, car 3.0-10, carData 3.0-4, caTools 1.18.2, colorspace 1.4-1, conquer 1.0.2, corrplot 0.89, cowplot 1.1.1, cpp11 0.2.7, cvAUC 1.1.0, data.table 1.14.0, devtools 2.4.1, dplyr 1.0.7, effectsize 0.4.5, emmeans 1.6.1, esc 0.5.1, estimability 1.3, exactRankTests 0.8-32, farver 2.0.3, forcats 0.5.1, gdata 2.18.0, generics 0.1.0, GenomeInfoDb 1.24.2, GenomicRanges 1.40.0, genefilter 1.70.0, ggeffects 1.1.0, ggplot2 3.3.4, ggpubr 0.4.0, ggrepel 0.9.1, ggsci 2.9, ggsignif 0.6.2, glue 1.4.2, gplots 3.1.1, gridExtra 2.3, gtable 0.3.0, gtools 3.8.2, gUtils 0.2.0, haven 2.4.1, hms 1.1.0, insight 0.14.1, IRanges 2.22.2, isoband 0.2.2, km.ci 0.5-2, KMsurv 0.1-5, labeling 0.3, lme4 1.1-27, lollipops 1.5.1, maptools 1.1-1, MatrixModels 0.5-0, maxstat 0.7-25, minqa 1.2.4, modelr 0.1.8, Munsell 0.5.0, mvtnorm 1.1-2, nloptr 1.2.2.2, numDeriv 2016.8-1.1, openxlsx 4.2.4, parameters 0.14.0, pbkrtest 0.5.1, performance 0.7.2, plyr 1.8.6, png 0.1-7, polynom1.4-0, pROC 1.17.0.1, progress 1.2.2, quantreg 5.86, qvalue 2.20.0, RColorBrewer 1.1-2, RcppEigen 0.3.3.7.0, readr 1.4.0, readxl 1.3.1, rematch 1.0.1, reshape2 1.4.4, rio 0.5.26, ROCR 1.0-11, rstatix 0.7.0, S4Vectors 0.26.1, scales 1.1.1, sjlabelled 1.1.8, sjmisc 2.8.7, sjPlot 2.8.8, sjstats 0.18.1, skitools 0.0.0.9000, sp 1.4-2, SparseM 1.81, statmod 1.4.34, stringi 1.6.2, survminer 0.4.8, survMisc 0.5.5, tidyr 1.1.3, tidyselect 1.1.0, viridisLite 0.3.0, xtable 1.8-4, XVector 0.28.0, zip 2.1.1, zoo1.8-8.

### Reporting summary

Further information on research design is available in the Nature Research Reporting Summary linked to this article.

## Supplementary information

Supplementary information

Description of Additional Supplementary Files

Supplementary Data 1

Reporting Summary

## Data Availability

Code and data for the analyses and figures are available in an interactive notebook here: https://gitlab.com/sanjanalab/circle. All data analyzed in this manuscript are publicly available and, for reproducibility, are also included in the GitLab repository. Source WES data for training and validation cohorts can be obtained from the respective studies^7,15–19,22,23^. Replication timing and epigenomic data were obtained from the ENCODE and Roadmap Epigenomics projects, respectively^62,63^.

## Code availability

Code to run the CIRCLE model is available here: https://gitlab.com/sanjanalab/circle.
