# Publisher Correction: Recurrent somatic mutations as predictors of immunotherapy response

**DOI:** 10.1038/s41467-022-32460-4

**Published:** 2022-08-05

**Authors:** Zoran Z. Gajic, Aditya Deshpande, Mateusz Legut, Marcin Imieliński, Neville E. Sanjana

**Affiliations:** 1 New York Genome Center, New York, NY 10013 USA; 2 Department of Biology, New York University, New York, NY 10002 USA; 3 Department of Neuroscience and Physiology, New York University School of Medicine, New York, NY 10016 USA; 4 Tri-institutional Ph.D. Program in Computational Biology and Medicine, New York, NY USA; 5 Department of Pathology and Laboratory Medicine, Englander Institute for Precision Medicine, Institute for Computational Biomedicine, and Meyer Cancer Center, Weill Cornell Medicine, New York, NY 10065 USA

**Keywords:** Cancer genetics, Predictive medicine

Correction to: *Nature Communications* 10.1038/s41467-022-31055-3, published online 08 July 2022

The original HTML version of this Article contained an error in Fig. 5, in which text in panels **a** and **d** was corrupted. This has been corrected in the HTML version of the Article. The PDF version was correct from the time of publication.

