# Supplementary material for: Recurrent somatic mutations as predictors of immunotherapy response
Source: Nat Commun. 2022 Jul 8;13:3938. doi: 10.1038/s41467-022-31055-3 (PMC9270330; doi:10.1038/s41467-022-31055-3)
Supplement: Supplementary file 1 — Supplementary information [file 41467_2022_31055_MOESM1_ESM.pdf]

Supplementary information

A robust predictor of immunotherapy response using whole exome sequencing

Zoran Z. Gajic[1,2,3], Aditya Deshpande[1,4,5], Mateusz Legut[1,2,3],
Marcin Imielinski[1,5,*], Neville E. Sanjana[1,2,3 *]

[1] New York Genome Center, New York, NY 10013, USA.

[2] Department of Biology, New York University, New York, NY 10002, USA.

[3] Department of Neuroscience and Physiology, New York University School of Medicine, New York, NY 10016, USA.

[4] Tri-institutional Ph.D. Program in Computational Biology and Medicine, New York, NY.

[5] Department of Pathology and Laboratory Medicine, Englander Institute for Precision Medicine, Institute for Computational Biomedicine, and Meyer Cancer Center, Weill Cornell Medicine, New York, NY 10065, USA.

*To whom correspondence should be addressed: Marcin Imielinski (mimielinski@nygenome.org) and Neville Sanjana (nsanjana@nygenome.org).

Supplementary Figures

Supplementary Figure 1. High and moderate mutational subtypes are enriched in responders.

Supplementary Figure 2. Age, tumor mutational burden and tumor type for predictive genes.

Supplementary Figure 3. Tumor specificity of immunotherapy predictive genes.

Supplementary Figure 4. Pathway-level mutational analysis of immunotherapy response.

Supplementary Figure 5. Additional analyses of the CIRCLE classifier and feature set.

Supplementary Figure 6. CIRCLE classifications do not predict survival in cancer patients from the TCGA PanCancer Atlas.

Supplementary Figure 7. Performance of the CIRCLE classifier on validation cohorts.

Supplementary Tables

Supplementary Table 1. Mutation calling pipelines for each cohort.

Supplementary Table 2. Studies included in the meta-analysis.

Supplementary Table 3. Mutational classes in each study.

Supplementary Table 4. Regression significant pathways.

Supplementary Table 5. Significant pathways after removal of duplicate pathways.

Supplementary Table 6. Significant pathways that overlap a CRISPR genome-scale screen.

Supplementary Table 7. CIRCLE logistic regression coefficients.

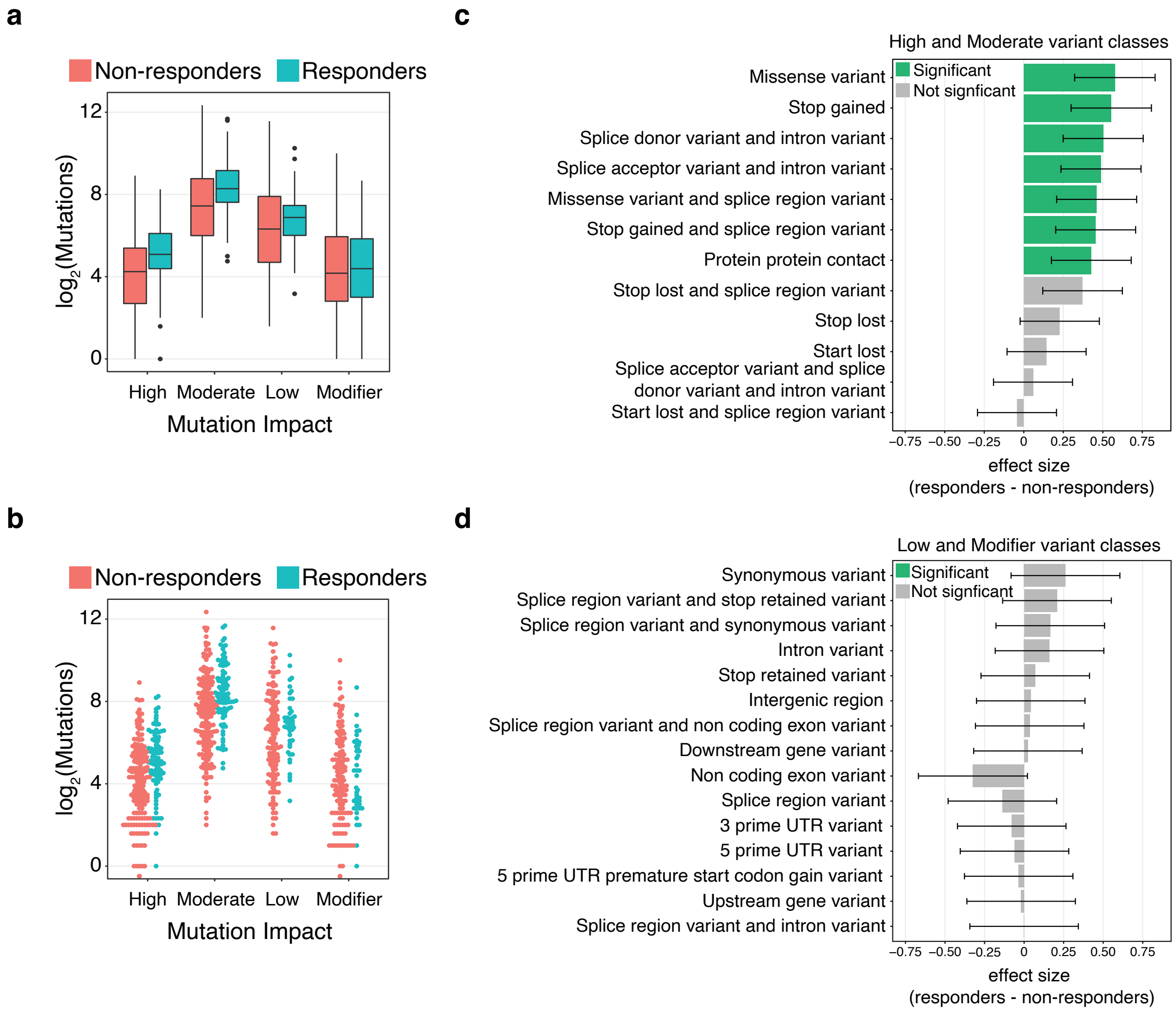

Supplementary Figure 1. High and moderate mutational subtypes are enriched in responders.

(a – b) Boxplot (**a**) and beeswarm plot (**b**) of the number of mutations per patient ($n = 272$ patients) for High, Moderate, Low, and Modifier mutations. The middle line of the box indicates the 50th percentile of data, box boundaries are defined as the 25th, and 75th percentile of the data, and whiskers are defined as the more extreme of the maximum/minimum value or 1.5 times the interquartile range.

(c - d) Standardized difference of means (*g*-statistic) of $\log_2$ TMB per patient ($n = 272$ patients) between responders and non-responders for the *High* and *Moderate* (**c**) and *Low* and *Modifier* (**d**) SnpEff mutation categories. Significance was determined using a two-sided Welch's t-test with Bonferroni correction. Significant mutation categories are shown in *green* and non-significant categories are shown in *grey*. Error bars indicate the 95% confidence interval of each effect size.

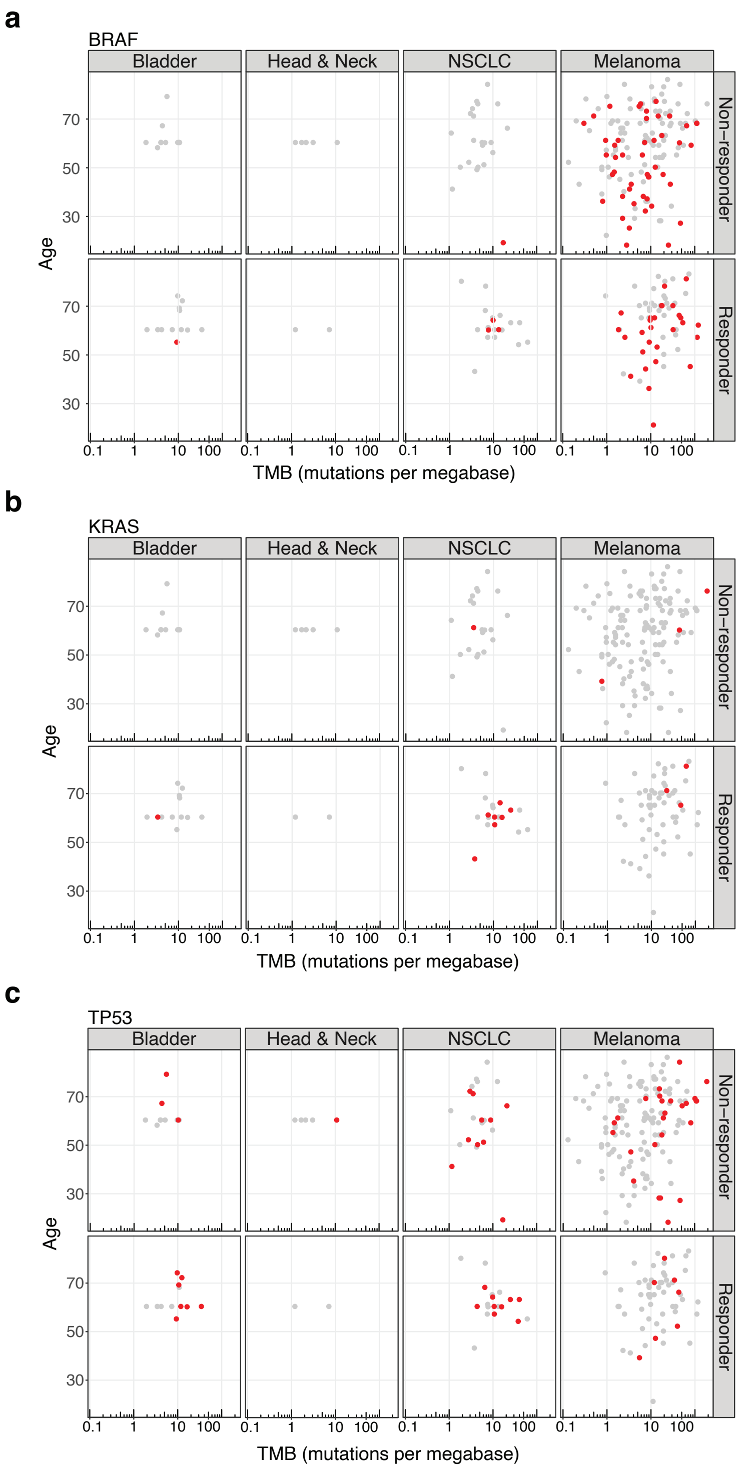

Supplementary Figure 2. Age, tumor mutational burden and tumor type for predictive genes.

(a - c) *BRAF* (**a**), *KRAS* (**b**), and *TP53* (**c**) mutations in the combined cohort with age, TMB and tumor type of the patients where they were identified. Patients with mutations in these genes are highlighted in *red*.

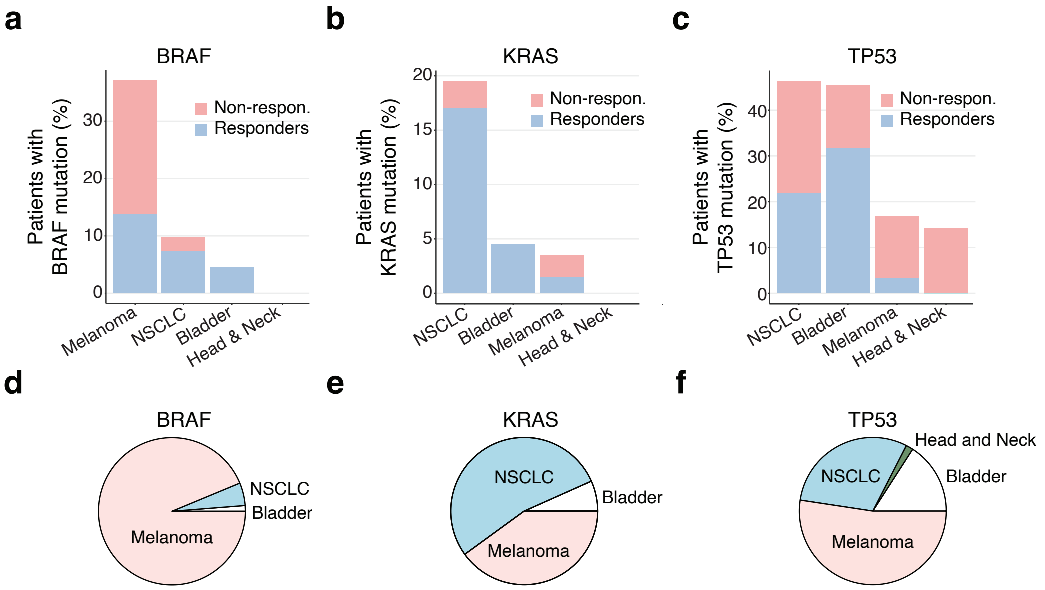

Supplementary Figure 3. Tumor specificity of immunotherapy predictive genes.

(a - c) Prevalence of *BRAF* (**a**), *KRAS* (**b**), and *TP53* (**c**) mutations in responders (*blue*) and non-responders (*red*) across the four tumor types.

(d - f) Distribution of *BRAF* (**d**), *KRAS* (**e**), and *TP53* (**f**) mutations across patients in the immunotherapy cohort.

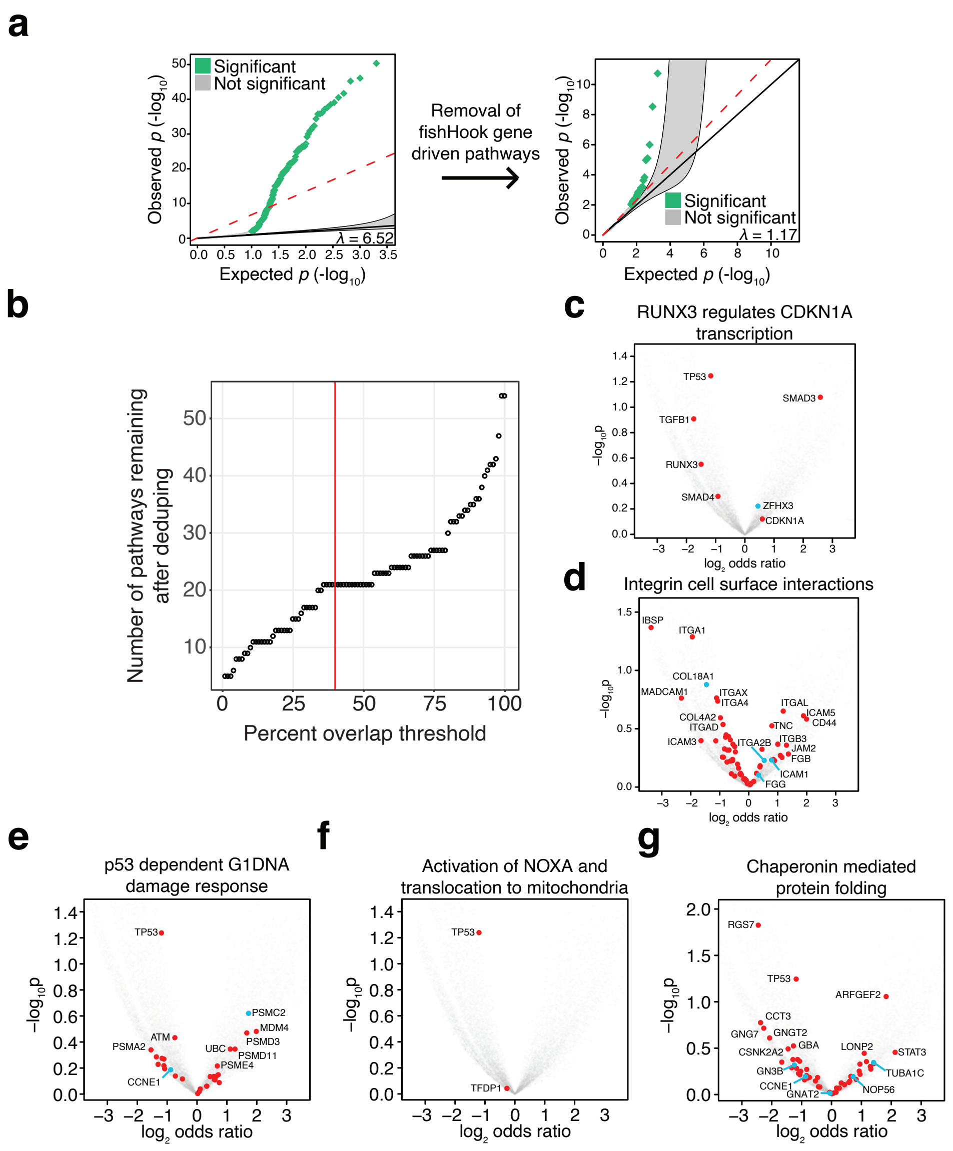

Supplementary Figure 4. Pathway-level mutational analysis of immunotherapy response.

(a) Quantile-quantile plot of fishHook nominated pathways before and after removal of pathway signals driven by the significant genes. The red dashed line indicates the slope of the regression line (λ). The shaded area indicates the 95% confidence interval for the null distribution. The *p*-values were obtained by comparing observed mutational rate to the right tail (one-sided) of the expected mutational rates derived from a gamma-Poisson model of genome-wide mutational density and the covariates replication timing, epigenetic state, and sequence context.

(b) In order to select an appropriate threshold for pathway de-duplication (due to gene overlap), we ordered the nominated pathways by significance and excluded pathways that shared a percentage of genes greater than 40%.

(c – g) Volcano plots of genes within five of the predictive pathways: *RUNX3 Regulates CDKN1A Transcription* (**c**), *Integrin Cell Surface Interactions* (**d**), *p53 Dependent G1 DNA Damage*

Response (**e**), *Activation of NOXA and Translocation to Mitochondria* (**f**), and *Chaperonin Mediated Protein Folding* (**g**). *Blue* highlighting indicates genes that were also found in the CRISPR screen. The *p*-values were obtained by comparing observed mutational rate to the right tail (one-sided) of the expected mutational rates derived from a gamma-Poisson model of genome-wide mutational density and the covariates replication timing, epigenetic state, and sequence context.

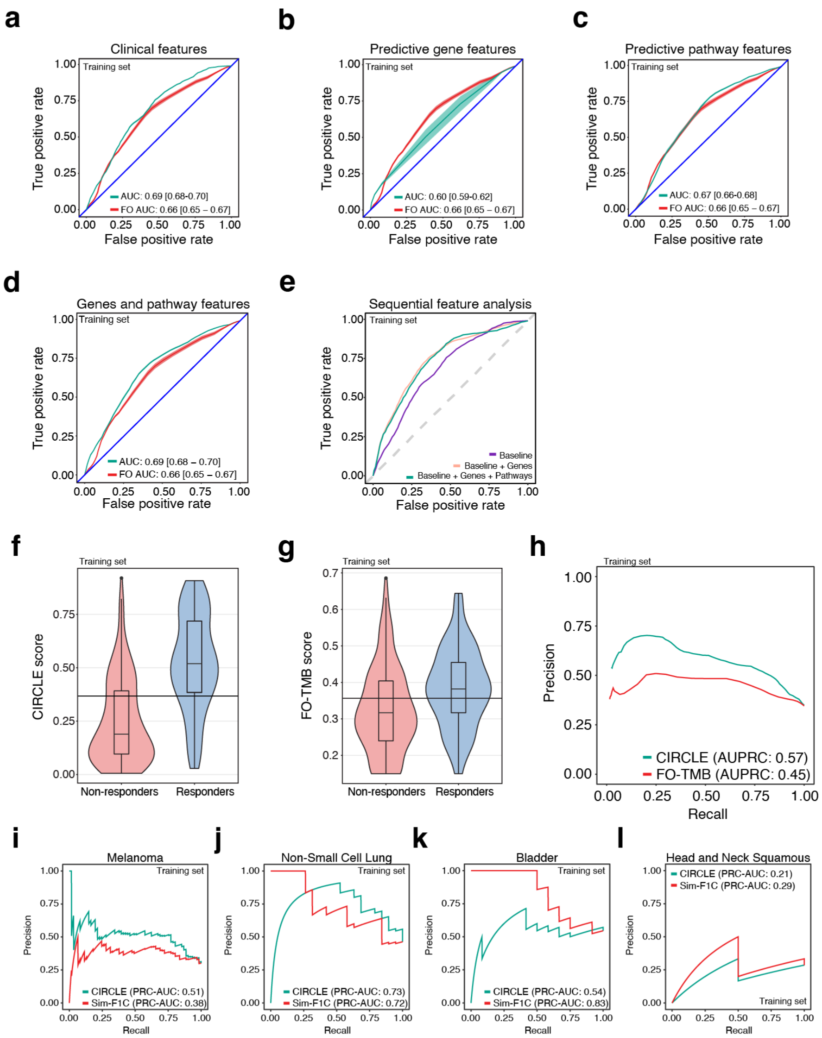

Supplementary Figure 5. Additional analyses of the CIRCLE classifier and feature set.

(a - d) Mean receiver-operator characteristic (ROC) curves for each of the tested sub-CIRCLE feature sets: clinical features (**a**), predictive genes (**b**), predictive pathways (**c**), predictive genes and pathways (**d**). Error bars indicate the standard deviation over 100 Monte-Carlo cross validation iterations.

(e) Mean ROC curves for sequentially tested feature sets including Clinical features only (*purple*), Clinical features and Genes (*beige*), and Clinical features, Genes, and Pathways – CIRCLE (*blue*).

(f - g) Distribution of CIRCLE (**f**), and FO-TMB scores (**g**) for true responders (*blue*, n = 94 patients) and true non-responders (*red*, n = 178 patients). The horizontal black line indicates the optimized threshold chosen from ROC analysis. The middle line of the box indicates the 50th percentile of data, box boundaries are defined as the 25th, and 75th percentile of the data, and whiskers are defined as the more extreme of the maximum/minimum value or 1.5 times the interquartile range.

(h) Mean precision recall curves for CIRCLE (*blue*) and FO-TMB (*red*) across the 100 Monte-Carlo cross validation iterations.

(**i-l**) Precision recall curves for CIRCLE (*blue*) and FO-TMB (*red*) in melanoma (**i**), NSCLC (**j**), bladder cancer (**k**), and head and neck squamous cancer (**l**).

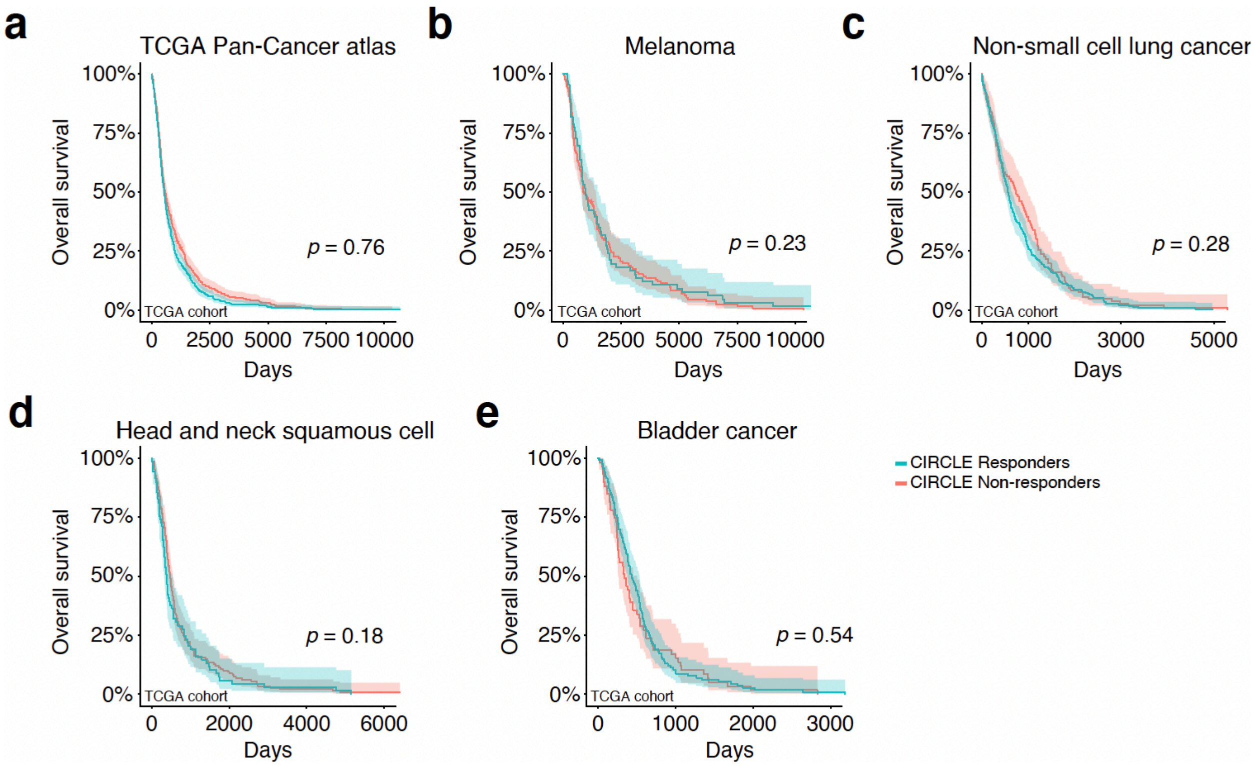

Supplementary Figure 6. CIRCLE classifications do not predict survival in cancer patients from the TCGA PanCancer Atlas.

(a - e) Kaplan-Meier plots TCGA PanCancer atlas patients classified as either CIRCLE responders or non-responders in the a cohort containing melanoma, NSLCLC, head and neck squamous cell and bladder cancer patients **(a)**, melanoma patients only **(b)**, NSCLC patients only **(c)**, head and neck squamous cell patients only **(d)**, and bladder cancer patients only **(e)**. Shaded areas indicate the 95% confidence interval. The *p*-values were calculated using a two-sided Cox proportional hazards test with tumor type, age, stage, and *TP53* mutational status as covariates.

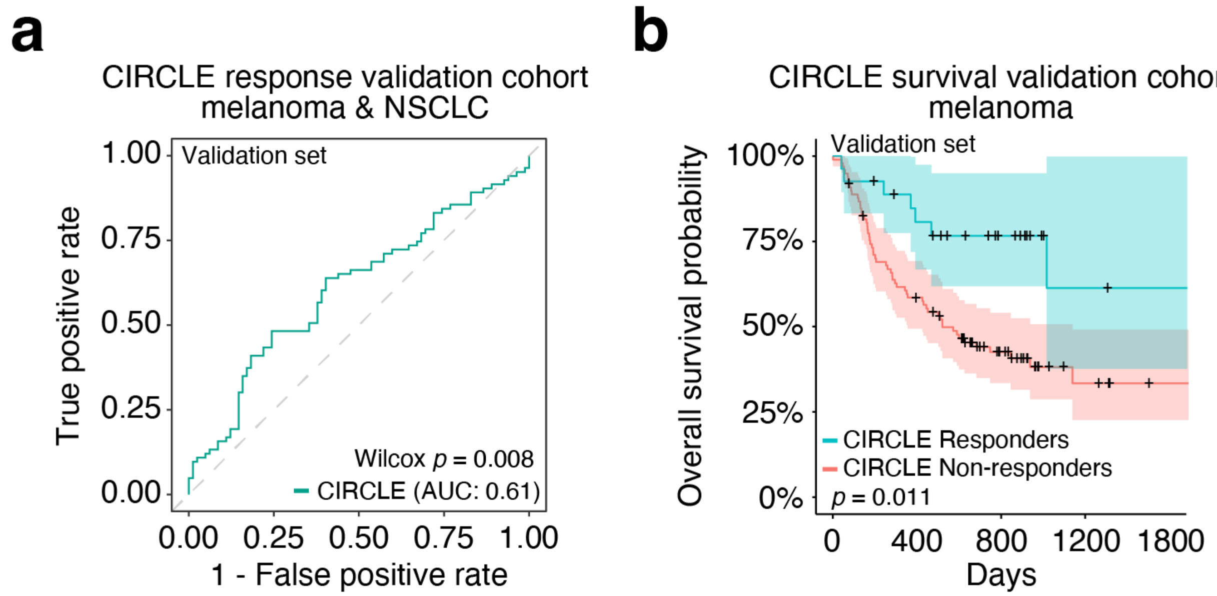

Supplementary Figure 7. Performance of the CIRCLE classifier on validation cohorts.

(a) Receiver-operator characteristic (ROC) curve for the CIRCLE model on a novel validation cohort containing melanoma ($n = 124$) and non-small cell lung cancer ($n = 41$) WES with matched ICB response status. Significance was calculated using a two-sided Wilxocon test.

(b) Kaplan-Meier plot of overall survival for validation patients classified as CIRCLE responders versus CIRCLE non-responders. Shaded areas indicate the 95% confidence interval. Statistical significance was calculated using a two-sided Cox proportional hazards test with Tumor Type (melanoma/non-small cell lung cancer) as a covariate.

Supplementary Table 1. Mutation calling pipelines for each cohort.

Study	Capture strategy	Sequencing Platform	Aligner	Mutation caller (SNV)	Quality control
Snyder et al. 2014	Agilent (Exon 44 or 51MB)	HiSeq 2000	BWA	Somatic Sniper	GATK
Rizvi et al. 2015	Agilent (Exon 44)	HiSeq 2000	BWA	Somatic Sniper, Varscan, Strelka, MuTect	GATK
Van Allen et al. 2015	Agilent V2 (44MB)	HiSeq 2000	Picard	MuTect	FireHose QC
Hugo et al. 2016	*Not given*	HiSeq 2000	NovoAlign	UG, Varscan2, MuTect, Oncotator	GATK, Samtools, Picard
Roh et al. 2017	Agilent Sure Select XT	HiSeq 2000/2500	BWA	MuTect	Picard, GATk
Miao et al. 2018	Agilent V2 (44MB)	HiSeq 2000	Picard	MuTect	FireHose QC
Hellman et al. 2019	Agilent (Exon 44 or 51MB)	HiSeq 2000/2500/4000	BWA	MuTect	Picard, GATK
Liu et al. 2019	Illumina	HiSeq 2000/2500	Picard	MuTect	FireHose QC

Supplementary Table 2. Studies included in the meta-analysis.

Cohort	Number of Patients	Complete Response (%)	Partial Response (%)	Stable Disease (%)	Progressive Disease (%)	Age (Average)	Male (%)	Female (%)	Mutations Per Eligible MB (Average)	Tumor Type(s)
Snyder et al., 2014	32	0	56	0	44	60	72	28	20.8	Melanoma
Van Allen et al., 2015	105	3	13	11	72	58	71	29	25.4	Melanoma
Rizvi et al., 2015	33	0	36	30	33	62	45	55	8.9	Lung
Hugo et al., 2016	38	18	37	0	45	61	71	29	21.5	Melanoma, Lung
Roh et al., 2017: anti-CTLA4 cohort	19	0	11	0	89	59	63	37	42.5	Melanoma
Roh et al., 2017: anti-PD1 cohort	14	0	0	14	86	52	57	43	38.8	Melanoma
Miao et at., 2018	78	5	26	29	40	63	56	44	11.7	Bladder, Lung, Head and Neck Squamous, Melanoma

Numbers in this table are rounded to the nearest whole or tenth of a number and thus percentiles may not sum to 100.

Supplementary Table 3. Mutational classes in each study.

Cohort	Number of Patients in Study	Total High Mutations	High Mutations per Patient	Total Moderate Mutations	Moderate Mutations per Patient	Total Low Mutations	Low Mutations per Patient	Total Modifier Mutations	Modifier Mutations per Patient	Included in Low and Modifier Analysis
Snyder et al., 2014	32	1648	51.50	18620	581.88	47	1.47	20	0.62	No
Rizvi et al., 2015	23	832	36.17	8069	350.83	15	0.65	11	0.48	No
Van Allen et at., 2015	93	4249	45.69	43297	465.56	24525	263.71	9311	100.12	Yes
Hugo et al., 2016	38	2114	55.63	22264	585.89	211	5.55	364	9.58	No
Roh et al., 2017: anti-CTLA4 cohort	19	1390	73.16	14168	745.68	8211	432.16	859	45.21	Yes
Roh et al., 2017: anti-PD1 cohort	12	862	71.83	9609	800.75	5466	455.50	625	52.08	Yes
Miao et at., 2018	55	2121	38.56	17226	313.20	7709	140.16	810	14.73	Yes

Supplementary Table 4. Regression significant pathways.

Pathway	Logistic Coefficient	OR	2.5 % CI	97.5 % CI	p-value	Standard Error
MAP2K and MAPK activation	1.6580	5.2470	1.9630	16.4730	0.0020	0.5360
Paradoxical activation of RAF signaling by kinase inactive BRAF	1.5730	4.8210	1.8370	14.7240	0.0030	0.5240
RAF activation	1.4310	4.1830	1.7090	11.3870	0.0030	0.4790
Signaling by FGFR2	2.0230	7.5630	2.3870	31.1610	0.0020	0.6430
Signaling by high-kinase activity BRAF mutants	1.6950	5.4480	2.0490	17.0230	0.0010	0.5330
Signaling by moderate kinase activity BRAF mutants	1.5730	4.8210	1.8370	14.7240	0.0030	0.5240
DNA Damage/Telomere Stress Induced Senescence	-1.1630	0.3130	0.1370	0.6770	0.0040	0.4060
Negative regulation of MAPK pathway	1.2010	3.3220	1.4080	8.4970	0.0080	0.4550
Signaling by FGFR3	1.3000	3.6690	1.4630	10.3540	0.0080	0.4940
Signaling by FGFR4	1.3040	3.6830	1.4580	10.4550	0.0090	0.4970
Negative feedback regulation of MAPK pathway	0.9700	2.6380	1.2710	5.6230	0.0100	0.3780
Signaling by FGFR	1.6350	5.1320	1.5510	21.4310	0.0130	0.6590
p53-Dependent G1 DNA Damage Response	-0.9450	0.3890	0.1770	0.8210	0.0150	0.3900
p53-Dependent G1/S DNA damage checkpoint	-0.9450	0.3890	0.1770	0.8210	0.0150	0.3900
Activated NTRK2 signals through FRS2 and FRS3	0.8220	2.2760	1.1270	4.6980	0.0230	0.3620
Activated NTRK2 signals through RAS	0.8060	2.2380	1.1070	4.6250	0.0260	0.3630
Integrin cell surface interactions	1.2780	3.5890	1.2540	11.1810	0.0210	0.5530
Signaling by NTRK2 (TRKB)	0.8580	2.3580	1.1150	5.1410	0.0270	0.3880
Signaling by PDGF	1.1940	3.2990	1.2270	9.8590	0.0230	0.5260
Stabilization of p53	-0.8450	0.4300	0.1980	0.8970	0.0280	0.3830
TP53 Regulates Transcription of Cell Death Genes	-0.8970	0.4080	0.1800	0.8840	0.0270	0.4050
TP53 regulates transcription of several additional cell death genes whose specific roles in p53-dependent apoptosis remain uncertain	-0.8660	0.4210	0.1940	0.8750	0.0240	0.3830
Transcriptional activation of cell cycle inhibitor p21	-0.9770	0.3760	0.1600	0.8430	0.0210	0.4220
Transcriptional activation of p53 responsive genes	-0.9770	0.3760	0.1600	0.8430	0.0210	0.4220
Signalling to ERKs	1.0100	2.7460	1.1370	7.1700	0.0300	0.4660
TP53 Regulates Transcription of Caspase Activators and Caspases	-0.8500	0.4280	0.1900	0.9190	0.0340	0.4000
Formation of Senescence-Associated Heterochromatin Foci (SAHF)	-0.7730	0.4620	0.2160	0.9520	0.0410	0.3770

RUNX3 regulates CDKN1A transcription	-0.7920	0.4530	0.2090	0.9470	0.0390	0.3840
Signaling by NTRKs	1.1900	3.2880	1.0910	11.6290	0.0450	0.5950
Signaling by FGFR1	0.9780	2.6590	1.0480	7.4080	0.0480	0.4940
Activation of NMDA receptor and postsynaptic events	0.9000	2.4600	1.0100	6.3610	0.0530	0.4660
VEGFA-VEGFR2 Pathway	0.9290	2.5320	1.0080	6.8080	0.0550	0.4840
Activation of NOXA and translocation to mitochondria	-0.7770	0.4600	0.2010	1.0100	0.0590	0.4110
Activation of PUMA and translocation to mitochondria	-0.7110	0.4910	0.2260	1.0300	0.0660	0.3860
Assembly of collagen fibrils and other multimeric structures	1.0860	2.9640	0.9910	9.7910	0.0600	0.5770
CD28 dependent Vav1 pathway	-0.7640	0.4660	0.2020	1.0340	0.0660	0.4150
Cell surface interactions at the vascular wall	0.9690	2.6360	0.9820	7.7030	0.0630	0.5210
Signaling by FGFR2 in disease	0.6900	1.9940	0.9630	4.2120	0.0650	0.3750
TP53 Regulates Transcription of Genes Involved in G1 Cell Cycle Arrest	-0.7160	0.4890	0.2220	1.0400	0.0690	0.3930
Chaperonin-mediated protein folding	-0.7700	0.4630	0.1930	1.0700	0.0770	0.4350
Collagen chain trimerization	1.0180	2.7670	0.9150	9.2410	0.0810	0.5830
Collagen formation	1.1580	3.1830	0.9510	12.8240	0.0750	0.6510
PI5P Regulates TP53 Acetylation	-0.7080	0.4930	0.2190	1.0660	0.0780	0.4020
Regulation of TP53 Expression	-0.7360	0.4790	0.2080	1.0580	0.0750	0.4130
Scavenging by Class A Receptors	-0.7380	0.4780	0.2050	1.0820	0.0810	0.4230
Autodegradation of the E3 ubiquitin ligase COP1	-0.6550	0.5200	0.2430	1.0760	0.0840	0.3780
FMO oxidises nucleophiles	0.7450	2.1060	0.8970	5.0320	0.0890	0.4380
Protein folding	-0.7530	0.4710	0.1930	1.1130	0.0910	0.4450
Regulation of TP53 Activity through Association with Co-factors	-0.6550	0.5200	0.2420	1.0790	0.0850	0.3810
Signaling by BRAF and RAF fusions	0.8910	2.4380	0.8990	7.3350	0.0920	0.5300
Signaling by NTRK1 (TRKA)	0.8980	2.4550	0.9040	7.4310	0.0910	0.5310
Collagen biosynthesis and modifying enzymes	1.0160	2.7630	0.8620	10.0400	0.1000	0.6170
Signaling by the B Cell Receptor (BCR)	0.7650	2.1500	0.8760	5.5200	0.1010	0.4660
Signaling by FGFR4 in disease	0.5700	1.7690	0.8890	3.5530	0.1050	0.3520

Pathway p-values were calculated as described in the Gene nomination section of the Methods.

Supplementary Table 5. Significant pathways after removal of duplicate pathways.

Pathway	Logistic Coefficient	OR	2.5 % CI	97.5 % CI	p-value	Standard Error
MAP2K and MAPK activation	1.6580	5.2470	1.9630	16.4730	0.0020	0.5360
Signaling by FGFR2	2.0230	7.5630	2.3870	31.1610	0.0020	0.6430
DNA Damage/Telomere Stress Induced Senescence	-1.1630	0.3130	0.1370	0.6770	0.0040	0.4060
Negative regulation of MAPK pathway	1.2010	3.3220	1.4080	8.4970	0.0080	0.4550
p53-Dependent G1 DNA Damage Response	-0.9450	0.3890	0.1770	0.8210	0.0150	0.3900
Integrin cell surface interactions	1.2780	3.5890	1.2540	11.1810	0.0210	0.5530
Signaling by PDGF	1.1940	3.2990	1.2270	9.8590	0.0230	0.5260
TP53 Regulates Transcription of Cell Death Genes	-0.8970	0.4080	0.1800	0.8840	0.0270	0.4050
Signalling to ERKs	1.0100	2.7460	1.1370	7.1700	0.0300	0.4660
RUNX3 regulates CDKN1A transcription	-0.7920	0.4530	0.2090	0.9470	0.0390	0.3840
Activation of NMDA receptor and postsynaptic events	0.9000	2.4600	1.0100	6.3610	0.0530	0.4660
Activation of NOXA and translocation to mitochondria	-0.7770	0.4600	0.2010	1.0100	0.0590	0.4110
Assembly of collagen fibrils and other multimeric structures	1.0860	2.9640	0.9910	9.7910	0.0600	0.5770
CD28 dependent Vav1 pathway	-0.7640	0.4660	0.2020	1.0340	0.0660	0.4150
Cell surface interactions at the vascular wall	0.9690	2.6360	0.9820	7.7030	0.0630	0.5210
Chaperonin-mediated protein folding	-0.7700	0.4630	0.1930	1.0700	0.0770	0.4350
PI5P Regulates TP53 Acetylation	-0.7080	0.4930	0.2190	1.0660	0.0780	0.4020
Regulation of TP53 Expression	-0.7360	0.4790	0.2080	1.0580	0.0750	0.4130
Scavenging by Class A Receptors	-0.7380	0.4780	0.2050	1.0820	0.0810	0.4230
FMO oxidises nucleophiles	0.7450	2.1060	0.8970	5.0320	0.0890	0.4380
Regulation of TP53 Activity through Association with Co-factors	-0.6550	0.5200	0.2420	1.0790	0.0850	0.3810

Pathway p-values were calculated as described in the Gene nomination section of the Methods.

Supplementary Table 6. Significant pathways that overlap a CRISPR genome-scale screen.

Pathway	Number of Overlapping Genes	q-value	Overlapping Genes
p53-Dependent G1 DNA Damage Response	10	0.00026	PSMB5, PSMA6, PSMC2, PSMD7, PSMA5, UBA52, PSMB2, PSMA7, CCNE1, PSMB4
Activation of NOXA and translocation to mitochondria	2	0.00477	PMAIP1, E2F1
Scavenging by Class A Receptors	3	0.01882	CALR, APOE, COLEC12
Integrin cell surface interactions	6	0.02790	ICAM1, ITGA2B, FGG, ITGB1, COL18A1, VTN
Chaperonin-mediated protein folding	7	0.04908	GNB3, CSNK2B, TUBA1C, GNAT2, CCNE1, NOP56, TUBB2B
RUNX3 regulates CDKN1A transcription	1	0.05872	ZFHX3
MAP2K and MAPK activation	3	0.08057	BRAF, ITGA2B, FGG
DNA Damage/Telomere Stress Induced Senescence	4	0.10003	HIST1H2BO, CCNE1, HIST1H2BK, H2AFV
Regulation of TP53 Activity through Association with Co-factors	1	0.10872	AKT3
Signalling to ERKs	2	0.10872	CRKL, BRAF
Negative regulation of MAPK pathway	2	0.15750	BRAF, UBA52
TP53 Regulates Transcription of Cell Death Genes	2	0.18393	PMAIP1, CASP2
Signaling by FGFR2	3	0.20444	BRAF, FGF4, UBA52
Signaling by PDGF	2	0.20444	CRKL, STAT1
Assembly of collagen fibrils and other multimeric structures	2	0.22342	COL5A1, COL18A1
Activation of NMDA receptor and postsynaptic events	1	0.23980	BRAF
Cell surface interactions at the vascular wall	3	0.52534	CD58, ITGB1, TEK

Pathway q-values were calculated as using a one-sided hypergeometric test.

Supplementary Table 7. CIRCLE logistic regression coefficients.

CIRCLE Variable	Coefficient	Standard Error	z-score	p-value
Intercept	-5.21	1.29	-4.03	5.48e-05
BCLAF1	-1.65	0.56	-2.96	3.12e-03
BRAF	0.71	0.39	1.83	6.78e-02
KRAS	1.00	0.72	1.39	1.65e-01
TP53	0.19	1.05	0.19	8.53e-01
Activation of NOXA and translocation to mitochondria	-0.50	0.84	-0.59	5.55e-01
Chaperonin mediated protein folding	-0.11	0.41	-0.26	7.94e-01
Integrin cell surface interactions	1.02	0.48	2.12	3.39e-02
MAP2K and MAPK activation	1.38	0.48	2.89	3.84e-03
RUNX3 regulates CDKN1A transcription	0.21	0.56	0.37	7.10e-01
Scavenging by Class A Receptors	-0.73	0.38	-1.91	5.65e-02
p53 Dependent G1 DNA Damage Response	-0.84	0.42	-2.01	4.47e-02
Age	0.03	0.01	1.97	4.91e-02
TMB	0.46	0.17	2.81	4.97e-03
Tumor Type: Head and Neck Squamous	-0.54	1.04	-0.52	6.05e-01
Tumor Type: Lung	-0.86	0.64	-1.34	1.80e-01
Tumor Type: Melanoma	-2.22	0.63	-3.53	4.22e-04

p-values were calculated using a two-tailed Wald's test of logistic regression coefficients.
