# Supplementary material for: Recurrent somatic mutations as predictors of immunotherapy response
Source: Nat Commun. 2022 Jul 8;13:3938. doi: 10.1038/s41467-022-31055-3 (PMC9270330; doi:10.1038/s41467-022-31055-3)
Supplement: Supplementary file 2 — Description of Additional Supplementary Files [file 41467_2022_31055_MOESM2_ESM.pdf]

Description of Additional Supplementary Files

File Name: Supplementary Data 1
Description: A excel file containing access data and sample information for samples used to create the replication timing, and epigenetic covariates.
