# Supplementary material for: Recurrent somatic mutations as predictors of immunotherapy response
Source: Nat Commun. 2022 Jul 8;13:3938. doi: 10.1038/s41467-022-31055-3 (PMC9270330; doi:10.1038/s41467-022-31055-3)
Supplement: Supplementary file 4 — Reporting Summary [file 41467_2022_31055_MOESM4_ESM.pdf]

Corresponding author(s): Neville E. Sanjana and Marcin Imielinski

Last updated by author(s): Apr 23, 2022

Reporting Summary

Nature Research wishes to improve the reproducibility of the work that we publish. This form provides structure for consistency and transparency in reporting. For further information on Nature Research policies, see our Editorial Policies and the Editorial Policy Checklist.

Statistics

For all statistical analyses, confirm that the following items are present in the figure legend, table legend, main text, or Methods section.

n/a	Confirmed	
☐	☒	The exact sample size (*n*) for each experimental group/condition, given as a discrete number and unit of measurement
☒	☐	A statement on whether measurements were taken from distinct samples or whether the same sample was measured repeatedly
☐	☒	The statistical test(s) used AND whether they are one- or two-sided<br>*Only common tests should be described solely by name; describe more complex techniques in the Methods section.*
☐	☒	A description of all covariates tested
☐	☒	A description of any assumptions or corrections, such as tests of normality and adjustment for multiple comparisons
☐	☒	A full description of the statistical parameters including central tendency (e.g. means) or other basic estimates (e.g. regression coefficient) AND variation (e.g. standard deviation) or associated estimates of uncertainty (e.g. confidence intervals)
☐	☒	For null hypothesis testing, the test statistic (e.g. *F*, *t*, *r*) with confidence intervals, effect sizes, degrees of freedom and *P* value noted<br>*Give P values as exact values whenever suitable.*
☒	☐	For Bayesian analysis, information on the choice of priors and Markov chain Monte Carlo settings
☒	☐	For hierarchical and complex designs, identification of the appropriate level for tests and full reporting of outcomes
☐	☒	Estimates of effect sizes (e.g. Cohen's *d*, Pearson's *r*), indicating how they were calculated

Our web collection on statistics for biologists contains articles on many of the points above.

Software and code

Policy information about availability of computer code

Data collection

No new data was collected in this study.

Data analysis

Analysis was conducted in R 4.0.2 and the following packages were used: abind 1.4-5 , bayestestR 0.10.0, BiocGenerics 0.34.0, broom 0.7.6, car 3.0-10, carData 3.0-4, caTools 1.18.2, colorspace 1.4-1, conquer 1.0.2, corrplot 0.89, cowplot 1.1.1, cpp11 0.2.7, cvAUC 1.1.0, data.table 1.14.0, devtools 2.4.1, dplyr 1.0.7, effectsize 0.4.5, emmeans 1.6.1, esc 0.5.1, estimability 1.3, exactRankTests 0.8-32, farver 2.0.3, forcats 0.5.1, gdata 2.18.0, generics 0.1.0, GenomeInfoDb 1.24.2, GenomicRanges 1.40.0, genefilter 1.70.0, ggeffects 1.1.0, ggplot2 3.3.4, ggpubr 0.4.0, ggrepel 0.9.1, ggsci 2.9, ggsignif 0.6.2, glue 1.4.2, gplots 3.1.1, gridExtra 2.3, gtable 0.3.0, gtools 3.8.2, gUtils 0.2.0, haven 2.4.1, hms 1.1.0, insight 0.14.1, IRanges 2.22.2, isoband 0.2.2, km.ci 0.5-2, KMsurv 0.1-5, labeling 0.3, lme4 1.1-27, lollipops 1.5.1, maptools 1.1-1, MatrixModels 0.5-0, maxstat 0.7-25, minqa 1.2.4, modelr 0.1.8, Munsell 0.5.0, mvtnorm 1.1-2, nloptr 1.2.2.2, numDeriv 2016.8-1.1, openxlsx 4.2.4, parameters 0.14.0, pbkrtest 0.5.1, performance 0.7.2, plyr 1.8.6, png 0.1-7, polynom1.4-0, pROC 1.17.0.1, progress 1.2.2, quantreg 5.86, qvalue 2.20.0, RColorBrewer 1.1-2, RcppEigen 0.3.3.7.0, readr 1.4.0, readxl 1.3.1, rematch 1.0.1, reshape2 1.4.4, rio 0.5.26, ROCR 1.0-11, rstatix 0.7.0, S4Vectors 0.26.1, scales 1.1.1, sjlabelled 1.1.8, sjmisc 2.8.7, sjPlot 2.8.8, sjstats 0.18.1, skitools 0.0.0.9000 , sp 1.4-2, SparseM 1.81, statmod 1.4.34, stringi 1.6.2, survminer 0.4.8, survMisc 0.5.5, tidyr 1.1.3, tidyselect 1.1.0, viridisLite 0.3.0, xtable 1.8-4, XVector 0.28.0, zip 2.1.1, zoo1.8-8.

The modified version of the fishHook source code used in this analysis is included in the accompanying repository: https://gitlab.com/sanjanalab/circle . The most recent version of the fishHook R package can be obtained through its repository: https://github.com/mskilab/fishHook .

The source code for the analysis and a version of the manuscript with code and results as a jupyter notebook file within can be obtained from https://gitlab.com/sanjanalab/circle .

For manuscripts utilizing custom algorithms or software that are central to the research but not yet described in published literature, software must be made available to editors and reviewers. We strongly encourage code deposition in a community repository (e.g. GitHub). See the Nature Research guidelines for submitting code & software for further information.

Data

Policy information about availability of data

All manuscripts must include a data availability statement. This statement should provide the following information, where applicable:

- Accession codes, unique identifiers, or web links for publicly available datasets
- A list of figures that have associated raw data
- A description of any restrictions on data availability

Code and data for the analyses and figures are available in an interactive notebook here: https://gitlab.com/sanjanalab/circle. All data analyzed in this manuscript are publicly available and, for reproducibility, are also included in the GitLab repository. Source WES data for training and validation cohorts can be obtained from the respective studies (refs. 7, 15-19, 22, 23). Replication timing and epigenomic data were obtained from the ENCODE and Roadmap Epigenomics projects, respectively (refs. 62, 63).

Field-specific reporting

Please select the one below that is the best fit for your research. If you are not sure, read the appropriate sections before making your selection.

☒ Life sciences ☐ Behavioural & social sciences ☐ Ecological, evolutionary & environmental sciences

For a reference copy of the document with all sections, see nature.com/documents/nr-reporting-summary-flat.pdf

Life sciences study design

All studies must disclose on these points even when the disclosure is negative.

Sample size	No sample size calculations were performed. We used all available patients from studies where we were able to obtain whole-exome variant calls.
Data exclusions	For analyses using specific patient features, all patients with available feature data were used for the analysis. For survival analyses, we excluded patients without survival data. For model training, we excluded patients with a clinical RECIST classification of Stable Disease, retaining only those with Complete Response, Partial Response and Progressive Disease.
Replication	We utilized Monte-Carlo and traditional cross-validation of predictive models within our discovery cohort (4 previously published studies) and on a separate validation cohort (2 previously published studies).
Randomization	For each (previously published) dataset in the discovery and validation cohorts, patients were already randomized as they were participants in clinical trials. No other randomization was performed.
Blinding	Blinding was not possible as all patients were from previously published studies.

Reporting for specific materials, systems and methods

We require information from authors about some types of materials, experimental systems and methods used in many studies. Here, indicate whether each material, system or method listed is relevant to your study. If you are not sure if a list item applies to your research, read the appropriate section before selecting a response.

Materials & experimental systems

n/a	Involved in the study	
☒	☐	Antibodies
☒	☐	Eukaryotic cell lines
☒	☐	Palaeontology and archaeology
☒	☐	Animals and other organisms
☒	☐	Human research participants
☒	☐	Clinical data
☒	☐	Dual use research of concern

Methods

n/a	Involved in the study	
☒	☐	ChIP-seq
☒	☐	Flow cytometry
☒	☐	MRI-based neuroimaging
